# Acylglycerol Kinase Inhibition Restores Mitophagy and Alleviates Alzheimer's Disease Pathology

**DOI:** 10.1002/mco2.70863

**Published:** 2026-07-08

**Authors:** Wensheng Li, Xuan Yu, Cuiping Guo, Yuting Huang, Zhen Wei, Yi Liu, Jian‐Zhi Wang, Rong Liu, Weike Ji, Qiuhong Duan, Jing Wang, Guihua Wang, Xiaochuan Wang

**Affiliations:** ^1^ Department of Biochemistry and Molecular Biology School of Basic Medicine, Tongji Medical College Huazhong University of Science and Technology Wuhan Hubei China; ^2^ Co‐Innovation Center of Neurodegeneration Nantong University Nantong China; ^3^ Department of Pathophysiology School of Basic Medicine Tongji Medical College Huazhong University of Science and Technology Wuhan China; ^4^ GI Cancer Research Institute Tongji Hospital, Huazhong University of Science and Technology Wuhan China; ^5^ Department of Pathology Maternal and Child Health Hospital of Hubei Province Tongji Medical College, Huazhong University of Science and Technology Wuhan China; ^6^ Institutes of Biomedical Sciences School of Medicine Hubei Key Laboratory of Cognitive and Affective Disorders Jianghan University Wuhan China; ^7^ Department of Immunology School of Basic Medicine Tongji Medical College Huazhong University of Science and Technology Wuhan China

**Keywords:** Alzheimer's disease, acylglycerol kinase, ATPase family AAA domain containing 3A, mitophagy, translocase of the inner mitochondrial membrane 23

## Abstract

Mitophagy is a conserved cellular process that removes dysfunctional or excess mitochondria. Increasing evidence suggests that impaired mitophagy plays a crucial role in AD development. Promoting mitophagy has been shown to be protective in models of AD, representing an important target of Alzheimer's disease (AD). However, the molecular mechanisms underlying impaired mitophagy in AD are still elusive. Here, we provide evidence that highly expressed acylglycerol kinase (AGK), a mitochondrial lipid kinase associated with mitochondrial protein transport, glycolysis, and platelet formation, is a key mediator of mitophagy in AD. We found that AGK promoted the binding of ATPase family AAA domain containing 3A to translocase of the inner mitochondrial membrane 23 and sequentially increased mitochondrial import of PTEN‐induced putative kinase 1, leading to the decrease of mitophagy. Further investigations revealed that the AGK downregulation in neuronal cells and APP/PS1 mice enhanced mitophagy, increased mitochondrial membrane potential, decreased pathological Tau/Aβ and neuroinflammation, and alleviated cognitive dysfunctions in the mice. Altogether our findings indicate that AGK plays a critical role in mediating mitophagy defects in AD; furthermore, downregulation of AGK promotes mitophagy and the decrease of Aβ and pathological Tau, providing an encouraging therapeutic treatment for AD.

## Graphical abstract

AGK (acylglycerol kinase) is abnormally upregulated in AD patients and mouse models. By stabilizing the TOM–TIM complex, this excess AGK facilitates PINK1 translocation to the IMM and prevents its OMM accumulation, leading to mitophagy impairment. Strikingly, AGK downregulation reverses this mitophagy deficiency and Aβ/Tau pathology, as well as ameliorates cognitive decline in APP/PS1 mice, highlighting AGK as a potential therapeutic target in AD.

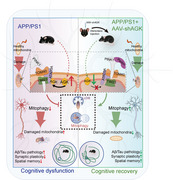

## 1 Introduction

Alzheimer's disease (AD) is the most widespread form of dementia characterized by progressive deterioration of memory. It encompasses age‐dependent accumulation of β‐amyloid (Aβ) and hyperphosphorylated tau (p‐Tau), mitochondrial abnormalities, and synaptic impairments [1, 2, 3]. Among these features, mitochondrial dysfunction has emerged as an early and critical event in AD pathogenesis, occurring even before overt plaque formation, suggesting that mitochondrial deficits may act as triggers rather than mere consequences of AD pathology [3]. Therefore, understanding the molecular mechanisms underlying mitochondrial dysfunction in AD is essential for developing effective therapeutic strategies.

Critically, impaired mitophagy drives the accumulation of mitochondria [4, 5], which promotes a vicious cycle with Aβ/Tau pathology, accelerating neurodegeneration [2, 6, 7, 8, 9]. Conversely, while inducing mitophagy to eliminate damaged mitochondria represents a promising therapeutic strategy for AD [10, 11, 12], Thus, maintaining mitochondrial quality control via mitophagy is vital to restrain AD. However, the underlying mechanisms require further elucidation.

Mitophagy is triggered by various signaling cues, such as oxidative stress, calcium overload, and membrane depolarization [4]. Driven by these cues, damaged mitochondria undergo macroautophagy followed by lysosomal degradation [13], thereby protecting cells from further damage and maintaining mitochondrial function under mild stress conditions. Although this process reduces the overall quantity of mitochondria, compensatory mechanism through mitochondrial biogenesis ensures an adequate mitochondrial pool and sustains cellular energy supply [14, 15, 16]. Crucially, the PTEN‐induced putative kinase 1 (PINK1)/Parkin cascade represents the canonical ubiquitin‐dependent pathway for this clearance. Key to this process is PINK1 stabilization on the outer mitochondrial membrane (OMM), regulated by mitochondrial protein import machinery including the ATPase family AAA domain containing 3A (ATAD3A), which bridges translocase of the OMM/translocase of the inner mitochondrial membrane (TOM/TIM) complexes [17, 18]. The translocase of the TOM complex assists in the assembly of PINK1 dimers, facilitating intermolecular phosphorylation of PINK1 molecules [19], which in turn recruits and phosphorylates PARKIN, activating its E3 ubiquitin ligase activity. PARKIN exhibits low substrate specificity and can ubiquitinate a wide range of OMM proteins [20], including CDGSH iron‐sulfur domain‐containing protein 1(CISD1), Translocase of outer mitochondrial membrane 20(TOM20), Voltage‐dependent anion channel 1 (VDAC1), Mitofusin(MFN) and others [21], ultimately activating mitophagy [22]. Novel research [23] has revealed that the proautophagic protein AMBRA1 was recruited to the OMM and reduced the interaction between PINK1 and ATAD3A. However, the precise molecular mechanism governing the interaction between ATAD3A and TIM23 remains poorly characterized.

Acylglycerol kinase (AGK) is a mitochondrial lipid kinase that plays a critical role not only in maintaining lipid metabolism stability but also in modulating mitochondrial protein transport, glycolysis, and platelet generation [24]. Our evidence suggests that AGK influences mitochondrial protein import dynamics, potentially through interactions with the mitochondrial import machinery. Given the importance of proper PINK1 import and stabilization for mitophagy initiation, we hypothesized that AGK modulates the ATAD3A/TIM23/TOM40 complex assembly, thereby affecting PINK1 transport and mitophagy efficiency. In this study, elevated expression of AGK was observed in the hippocampal tissues of AD patients and 13‐month‐old APP/PS1 mice. By transfecting neuronal cells and APP/PS1 mice with shRNA targeting AGK, we found that downregulation of AGK decreased the binding between ATAD3A and TIM23, thereby restoring PINK1 accumulation on the OMM and promoting mitophagy. Consequently, this treatment increased mitochondrial membrane potential, decreased levels of p‐Tau, increased dendritic spine numbers, and improved cognitive function in the mice. These findings provide novel insights into the role of AGK in regulating mitophagy, thereby offering a promising molecular target for therapeutic interventions in AD.

## 2 Results

### 2.1 High AGK Expression and PINK1 Deficiency Were Associated With AD

To identify genes linking age, mitochondrial function, and AD, we performed weighted gene coexpression network analysis (WGCNA) and Pearson correlation analysis in three brain regions: ventrolateral prefrontal cortex (VC), cingulate gyrus (CR), and prefrontal cortex (PFC). The top three age‐correlated modules (Figure S1A–C) were analyzed, leading to the identification of three key genes: AGK, ACACB, and CPT1A (Figure S1D). ACACB and CPT1A functions mainly in fatty acid metabolism and is classical metabolic enzymes. Given the diversity of AGK functions, this study focused primarily on AGK.

**FIGURE 1:**
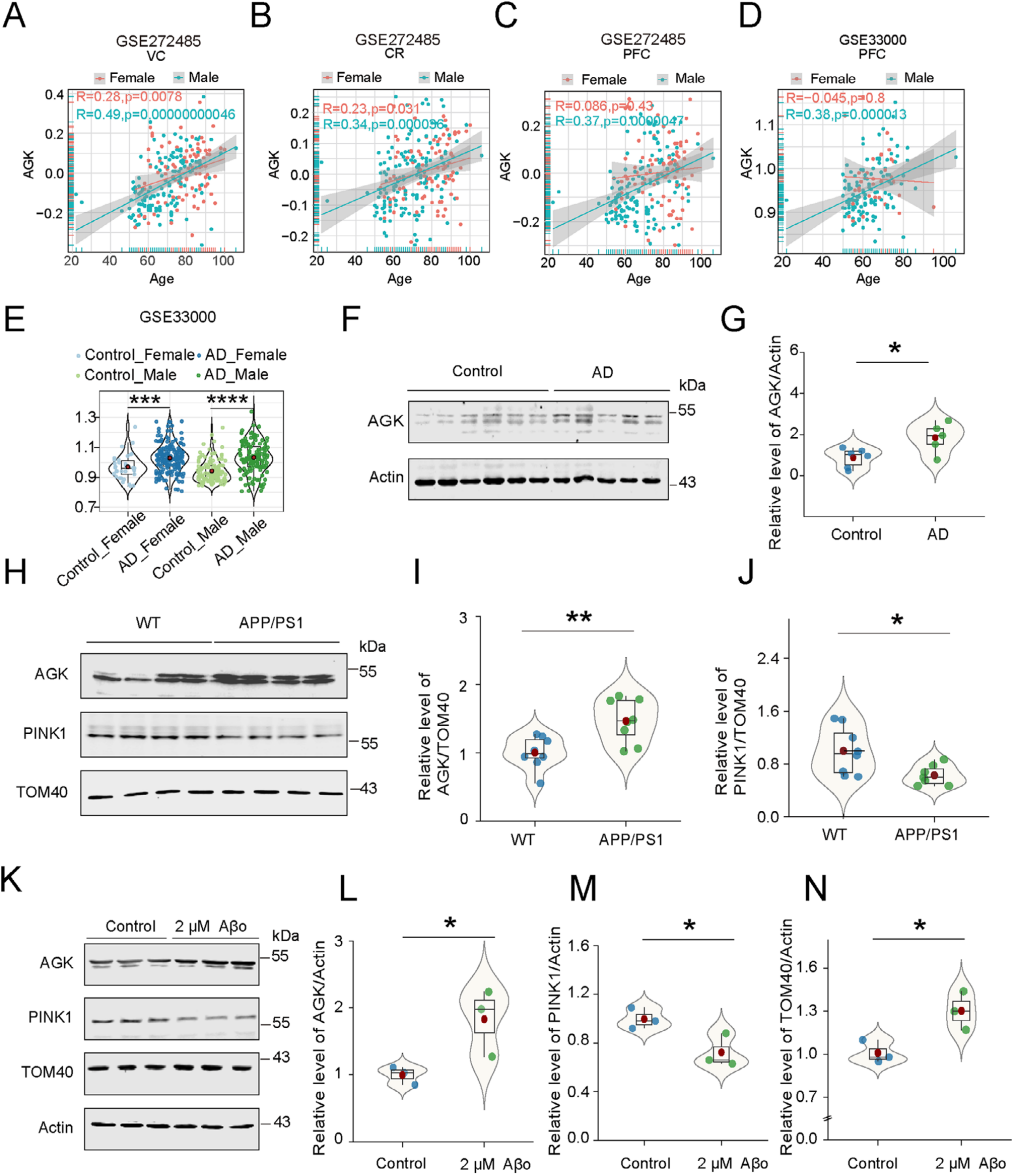
High AGK expression and PINK1 deficiency were associated with AD. (A–C) In the GSE272485 dataset, the correlation analysis between AGK expression levels and age in the VC, CR, and PFC. (D) In the GSE33000 dataset, the correlation analysis between AGK expression levels and age in the healthy group. (E) In the GSE33000 dataset, a comparison of AGK expression levels between control and AD groups across different genders. (F) The protein levels of AGK in the hippocampus of normal individuals and AD patients were examined. Detailed sample information can be found in Table S2. (G) Statistical analysis of the levels of AGK in the hippocampus between normal individuals and AD patients. The data were normalized to actin protein levels (*n* = 6, 5). (H) Mitochondria were isolated from the hippocampus of 13‐month‐old C57 and APP/PS1 mice, followed by WB to determine the levels of PINK1 and AGK. (I and J) Statistical analysis of the levels of mitochondrial AGK and PINK1 proteins in C57 and APP/PS1 mice. The data were normalized to the abundance of TOM40 protein (*n* = 8, 8). (K) The protein levels of PINK1, AGK, and TOM40 in primary neurons treated with 2 µM Aβ_1–42_ oligomers for 48 h. (L–N) Statistical analysis was conducted to evaluate the protein levels of AGK, PINK1, and TOM40 in primary neurons treated with Aβo. The data were normalized to actin (*n* = 3, 3). Data are presented as mean ± standard deviation (means ± SD); *p < 0.05, **p < 0.01 (two‐tailed Student's *t*‐test).

Analysis of AGK expression revealed significant positive correlations with age in VC, CR, and PFC from healthy humans (Figure 1A–D). Critically, AGK expression was significantly upregulated in AD patients versus controls across genders (Figure 1E), a result confirmed at the protein level by higher AGK in AD patient hippocampus (Figure 1F,G).

This dysregulation was recapitulated in experimental models. APP/PS1 mice exhibited increased mitochondrial AGK alongside decreased levels of PINK1 (Figure 1H–J). Treating primary neurons with Aβ1–42 oligomers (2 µM) similarly decreased PINK1 while increasing AGK and the mitochondrial protein TOM40 (Figure 1K–N). These results suggest that Aβ‐induced mitochondrial stress drives pathological AGK elevation and PINK1 reduction, the latter of which is intimately associated with impaired mitophagy.

### 2.2 AGK Deficiency Induced an Increase in PINK1 of Mitochondrion and Promotes Mitophagy

**FIGURE 2:**
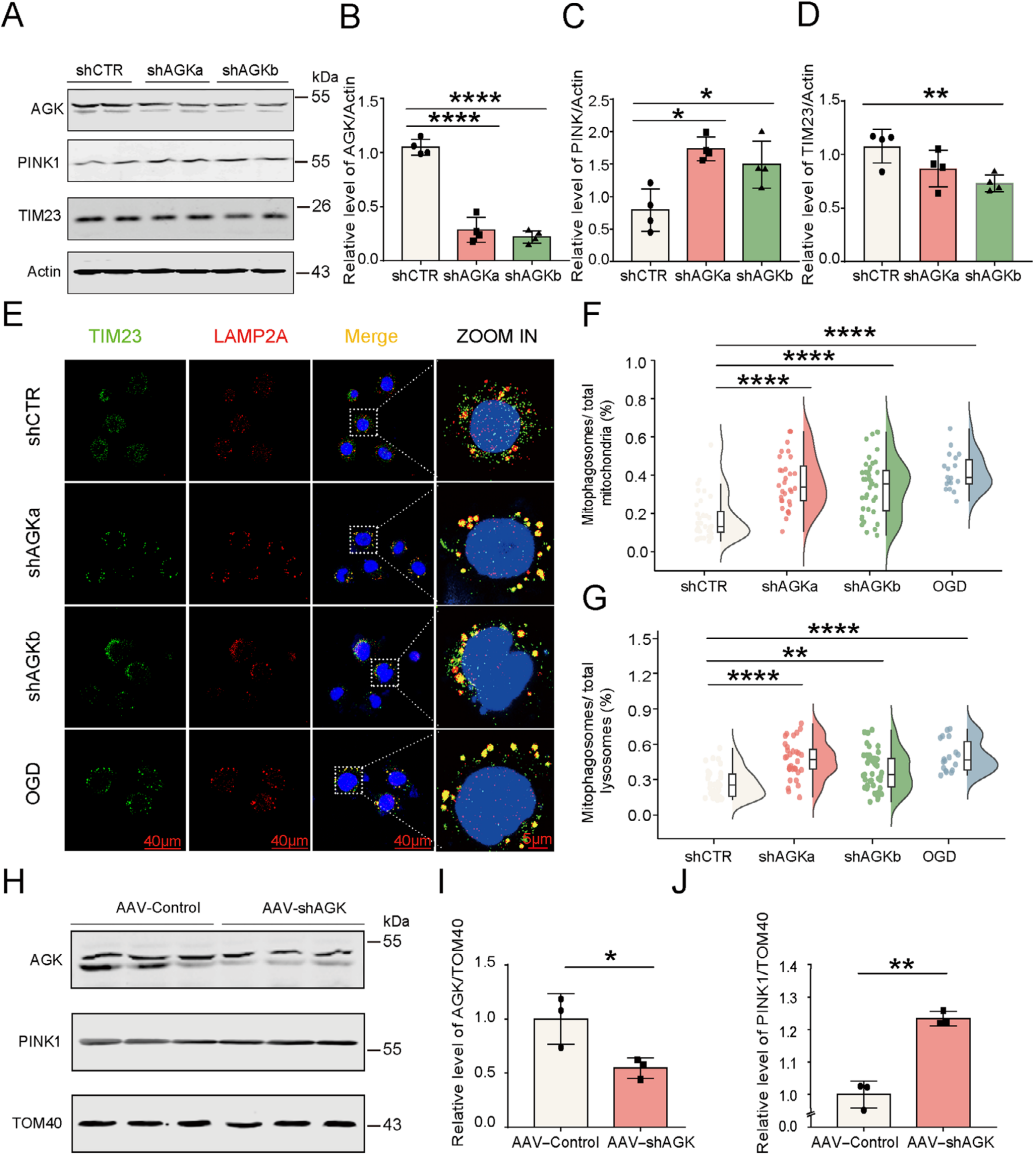
AGK deficiency induced an increase in PINK1 of mitochondrion and promotes mitophagy. (A) Protein levels of PINK1, TIM23, and AGK were detected using WB in SH‐SY5Y cell lines with AGK knockdown (shAGKa, shAGKb). (B–D) Statistical graphs representing the protein levels of AGK, PINK1, and TIM23, normalized to actin as in (A), *n* = 4 in each group. (E) Representative images of immunostainings showed the colocalization of TIM20 and LAMP2A in SH‐SY5Y cells with nontargeting shRNA (shCTR) or AGK‐specific shRNA, and the OGD treatment group. Green fluorescence represents the mitochondrial protein TIM23, while red fluorescence represents the lysosomal protein LAMP2A. Scale bar, 40 and 5 µm (zoom in). (F and G) Frequency of colocalization of TIM23 with LAMP2A (mitophagosomes) as in (E), *n* = 32, 32, 43, 18. (H) Mitochondria were extracted from the hippocampal tissues of 6‐month‐old APP/PS1 mice injected with AAV9–control or AAV9–shAGK, and protein levels of PINK1 and AGK were determined by immunoblotting. (I and J) Statistical graphs presented the protein levels of mitochondrial PINK1 and AGK, normalized to TOM40, *n* = 3 in each group. Data are presented as mean ± SD; *p < 0.05, **p < 0.01, ****p < 0.0001 (two‐tailed Student's *t*‐test).

Accumulation of PINK1 and subsequent recruitment of PARKIN to the OMM have been shown to trigger mitophagy [25, 26, 27]. To investigate if AGK participates in the PINK1–PARKIN signaling pathway, we first established Parkin‐overexpressing stable SH‐SY5Y cells (Figure S2A,B). Utilizing these cells, we further knocked down AGK via lentivirally delivered shRNA and selected for stable cell lines (Figure 2A,B). Subsequent immunoblotting analysis revealed that AGK knockdown significantly increased PINK1 levels while decreasing TIM23 levels (Figure 2C,D), suggesting a potential change in mitochondrial abundance linked to the PINK1 pathway. Additionally, immunofluorescence (IF) experiments revealed increased colocalization of mitochondrial protein TIM23 and lysosomal protein LAMP2A in AGK‐silenced cells, similar to oxygen glucose deprivation (OGD)‐treated cells, a commonly used mitophagy model (Figure 2E–G) [28, 29, 30]. To further validate the impact of AGK on mitochondrial quality and mitophagy, we labeled mitochondria with MitoTracker Red CMXRos and lysosomes with LAMP2A antibody. The results showed increased mitochondrial membrane potential and increased colocalization of mitochondria and lysosomes in the AGK knockdown group, while the AGK overexpression exhibited the opposite effects (Figure S3A–C). To demonstrate the impact of AGK on mitophagy, we quantified mitochondrial mass by measuring the ratio of mitochondrial DNA (mtDNA) to nuclear DNA by qPCR using total DNA extracted from SH‐SY5Y cells. The AGK silenced group exhibited a lower mtDNA copy number compared with the control group and HA‐AGK group (Figure S3F). These findings indicated that AGK deficiency reduced mitochondrial abundance but enhanced mitochondrial membrane potential. To further elucidate the role of AGK in mitophagy, stereotactic injection of AAV9–shAGK virus was performed in 6‐month‐old APP/PS1 mice, resulting in elevated levels of PINK1 in mitochondria (Figure 2H–J), indicating enhanced mitophagy signaling. In summary, these findings suggest that AGK acts as a potential modulator of mitophagy by regulating the mitochondrial PINK1 levels.

### 2.3 AGK Promotes the Formation of the ATAD3A/TIM23/TOM40 Complex and Facilitates PINK1 Transport Into Mitochondria

**FIGURE 3:**
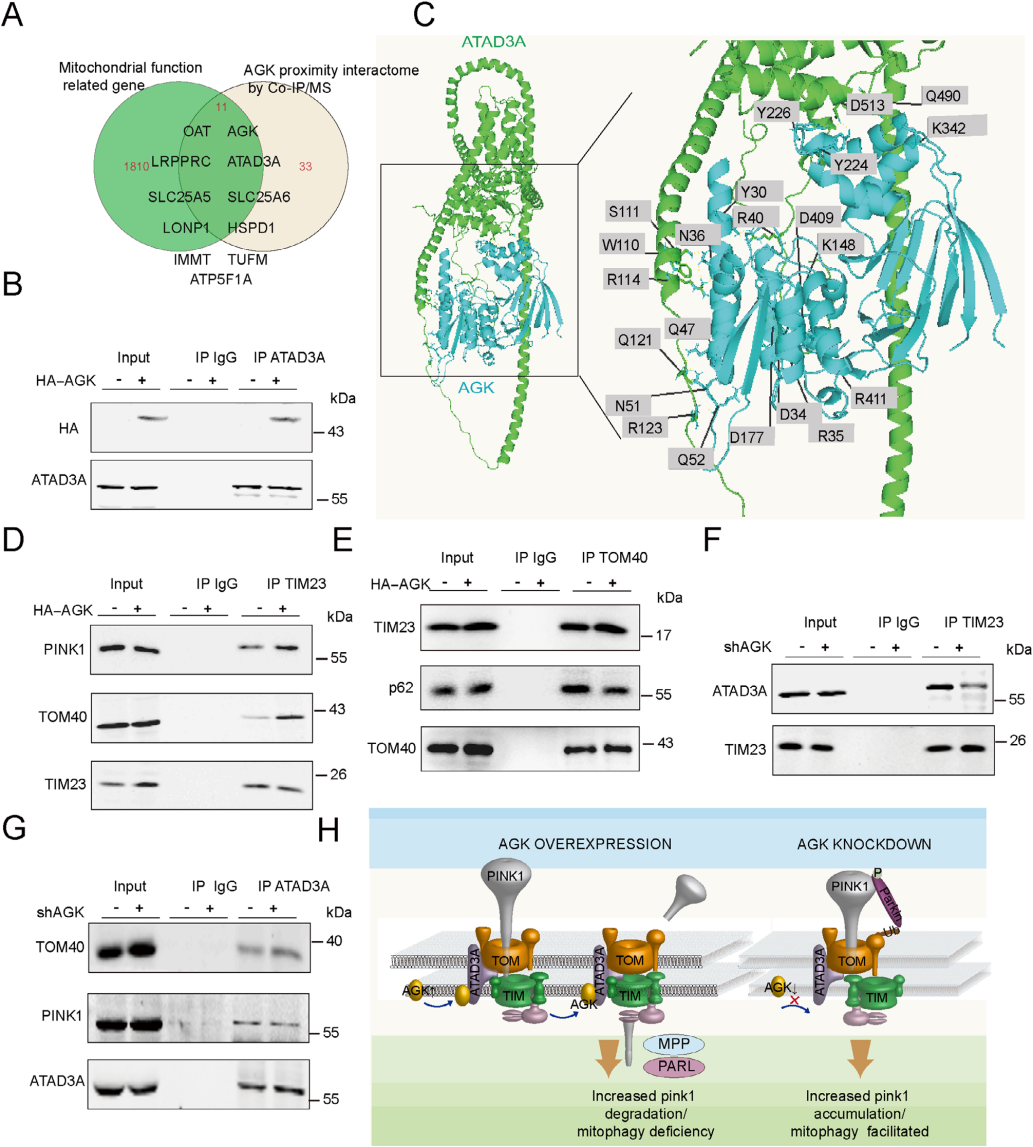
AGK promoted the formation of ATAD3A/TIM23/TOM40 complex and PINK1 influx on mitochondrion. (A) Venn diagram illustrates the overlap (11 shared candidates) between the mitochondrial function‐related gene set and the AGK proximity interactome identified by Co‐IP‐MS. (B) SH‐SY5Y cells overexpressing HA‐AGK were subjected to immunoprecipitation using anti‐ATAD3A antibody. The purified complexes were analyzed by immunoblotting using anti‐HA antibody. (C) Visualization of molecular docking of AGK and ATAD3A of the lowest energy, and a series of amino acid residues of the AGK–ATAD3A interaction interface. (D) SH‐SY5Y cells overexpressing AGK were immunoprecipitated using anti‐TIM23 antibody to capture the protein complexes. The purified complexes were subjected to protein immunoblot analysis using anti‐TOM40 and anti‐PINK1 antibodies. (E) SH‐SY5Y cells overexpressing AGK were subjected to immunoprecipitation using anti‐TOM40 antibody. Immunopurified complexes were analyzed by immunoblotting using anti‐TIM23 and anti‐SQSTM1/p62 antibody. (F) IP experiments were performed on stable cell lines with AGK knockdown using SH‐SY5Y cells. Protein extracts were subjected to immunoprecipitation using an anti‐TIM23 antibody. Immunopurified complexes were analyzed by immunoblotting using an anti‐ATAD3A antibody. (G) AGK‐silenced SH‐SY5Y cells were subjected to immunoprecipitation using an anti‐ATAD3A antibody. Immunopurified complexes were analyzed by immunoblotting using anti‐TOM40 and anti‐PINK1 antibodies. (H) Schematic diagram illustrating the proposed role of AGK in mitophagy deficiency via stabilizing the TOM–TIM complex, facilitating PINK1 translocation to the IMM and preventing its OMM accumulation.

To explore the mechanism of AGK in mitophagy, we performed integrative analysis of the AGK proximity interactome (Co‐IP/MS) and mitochondrial function‐related gene sets, which revealed 11 overlapping candidates (Figure 3A), including ATAD3A, a scaffold protein regulating mitophagy [22, 31]. Co‐IP assays in HA–AGK‐overexpressing SH‐SY5Y cells confirmed direct binding between AGK and ATAD3A (Figure 3B). Molecular docking further predicted critical interfacial residues stabilizing this interaction (Figure 3C). AGK strengthened interactions between TIM23 and TOM40, while simultaneously increasing PINK1 association with the complex (Figure 3D). Furthermore, TOM40 immunoprecipitates from HA–AGK group showed less binding to autophagy adaptor SQSTM1/p62 [32] (Figure 3E), revealing AGK bridges mitochondrial import machinery with autophagic clearance. Additionally, reciprocal experiments in AGK‐silenced cells demonstrated decrease of ATAD3A–TIM23 interaction (Figure 3F) and reduced PINK1–ATAD3A interaction (Figure 3G), indicating AGK knockdown disrupts ATAD3A/TIM23/TOM40 complex and reduced the transport of PINK1. These findings indicate that AGK facilitates the interaction between ATAD3A and TIM23, thereby promoting the transport of PINK1. In contrast, inhibition of AGK expression leads to the accumulation of PINK1, which is associated with mitophagy induction (Figure 3H).

### 2.4 Inhibition of AGK Blocked Abeta‐Induced Mitochondrial Dysfunction

**FIGURE 4:**
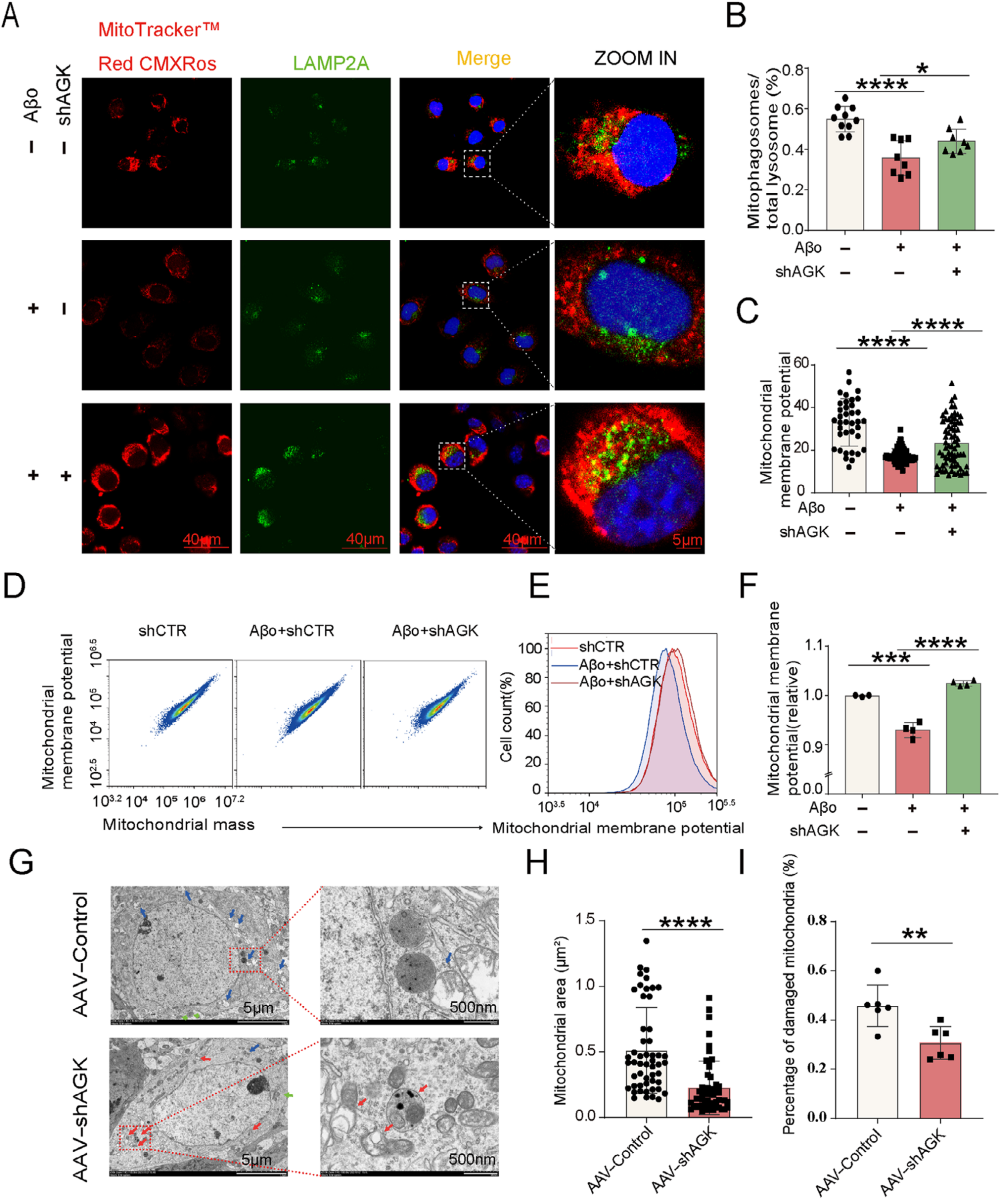
Inhibition of AGK blocked Abeta‐induced mitochondrial dysfunction. (A) Representative images of SH‐SY5Y cells expressing shCTR or shAGK with or without Aβ_1–42_ treatment, scale bar = 40 and 5 µm (zoom in). (B) Statistical analysis of mitophagosomes rate, as in (A), *n* = 10, 8, 8. (C) Statistical analysis of the average red fluorescence intensities, representing membrane potential, as in (A), *n* = 37, 70, 67. (D) Flow cytometry analysis of live cells costained with the two aforementioned dyes. (E) Mitochondrial membrane potential in flow cytometry analysis of shCTR, Aβo + shCTR, and Aβo + shAGK groups. (F) Statistical analysis of the average ratio between red and green fluorescence intensities, representing relative membrane potential, in SH‐SY5Y cells of shCTR, Aβo + shCTR, and Aβo + shAGK groups, *n* = 3, 4, 4. (G) Representative TEM images of the hippocampus from AAV‐injected APP/PS1 mice. Healthy mitochondria were indicated by green arrows, mitophagy by red arrows, and damaged mitochondria by blue arrows. Scale bar, 5 µm or 500 nm. (H and I) Mitochondrial area analysis and percentage analysis of damaged mitochondria for (G) (*n* = 6 slices from 3 mouse per group). The graph reports mean ± SD; *p < 0.05, **p < 0.01, ***p < 0.001, ****p < 0.0001 (two tailed Student's *t*‐test).

To assess the effects of AGK on mitochondrial status, we costained AGK overexpressed and silenced cells with MitoTracker Green FM and MitoTracker Red CMXRos whose fluorescence intensity positively correlated with mitochondrial membrane potential (Figure S3D). As assessed by the ratio of cellular membrane potential to mitochondrial mass, the AGK overexpression group showed reduced relative membrane potential, while the AGK silenced group displayed enhanced relative membrane potential (Figure S3E). This might achieve partial compensation for the loss of mitochondrial mass. Flow cytometry experiments (Figure S3G) confirmed that the AGK group exhibited a rightward shift in red fluorescence peak (Figure S3H), indicating a decrease in relative mitochondrial membrane potential (Figure S3I), which was consistent with the results from analysis of fluorescent images. Aβ oligomers can interfere with autophagosomes, impeding their transport to mature endo‐lysosomes [2, 8, 9]. Thus, we further treated SH‐SY5Y cells with Aβ_1–42_ oligomers and found that AGK depletion rescued the mitophagy impairment (Figure 4A–C) and restored relative mitochondrial membrane potential (Figure 4D–F). Similar findings were validated in N2A cells (Figure S4A–J). Consistently, transmission electron microscopy images revealed a reduction in total mitochondrial area and percentage of damaged mitochondria in the hippocampus of AAV9–shAGK‐treated APP/PS1 mice (Figure 4G–I).

### 2.5 AGK Downregulation Attenuates AD Pathology via Dual Modulation of Aβ/Tau Clearance and Neuroinflammation

**FIGURE 5:**
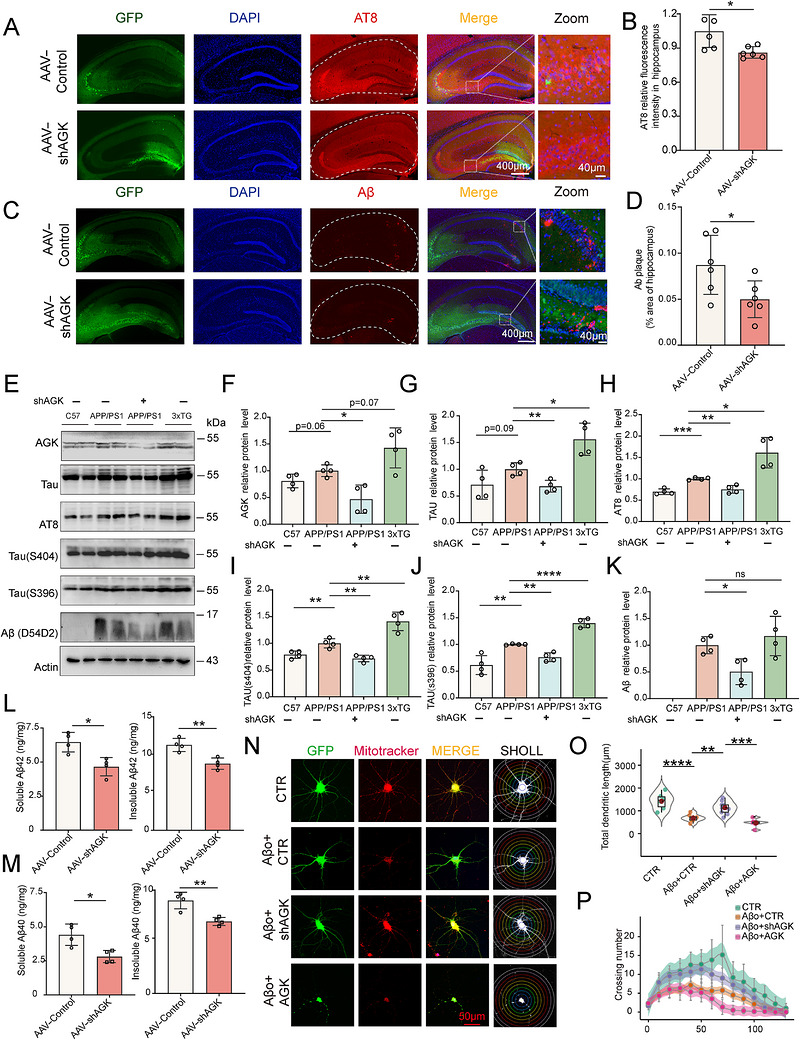
Downregulation of AGK improved AD pathology. (A and B) Immunofluorescence staining results for AT8 for APP/PS1 mice that underwent injection of AAV9–control and AAV9–shAGK. Scale bar = 400 and 40 µm (zoom in), *n* = 6, 6. (C and D) Immunofluorescence staining results for Aβ in APP/PS1 mice that underwent injection of AAV9–control and AAV9–shAGK. Scale bar = 400 and 40 µm (zoom in), *n* = 6, 6. (E) Hippocampal tissue samples were collected from APP/PS1 mice that underwent injection of AAV9–control and AAV9–shAGK were analyzed by immunoblotting, included wild‐type (C57) mice as a negative control and 3×TG mice as a positive control. (F–K) Statistical graphs representing the protein levels of AGK, total Tau, AT8, pS404, pS396, and Aβ normalized to actin as in (E), *n* = 4 per group. (L and M) The concentration of soluble and insoluble Aβ40 and Aβ42 were measured using an ELISA kit in both hippocampus of AAV–control and AAV–shAGK‐treated groups, *n* = 5 per group. (N) Mouse primary hippocampal neurons were treated with Aβ oligomers for 48 h, followed by transduction with AAV–GFP vector (control), AAV–GFP–shAGK, or AAV–GFP–AGK for 4 days. Representative images are shown. Scale bar = 50 µm. (O) Quantitative analyses of dendritic length of the neurons, *n* = 6, 8, 9, 6. (P) Sholl analysis of the neurons, *n* = 6, 8, 9, 6. The graph reports mean ± SD, *p < 0.05, **p < 0.01, ***p < 0.001, ****p < 0.0001 (two tailed Student's *t*‐test).

Enhanced mitophagy has been shown to improve neuronal mitochondrial function and reduce the accumulation of Aβ and tau abnormality [10, 23]. In our research, injection of AAV9–shAGK in APP/PS1 mice significantly reduced p‐Tau levels (Figure 5A,B) and Aβ plaque burden (Figures 5C,D and S5E) in hippocampus. Concurrently, AGK downregulation attenuated glial activation in APP/PS1 mice, which was characterized by a significant reduction in both Iba1^+^ microglial number (Figure S5A,B) and GFAP^+^ astrocytic number (Figure S5C,D), indicating suppressed neuroinflammation. Furthermore, Western blot analysis of hippocampal tissues confirmed marked reductions in total tau, p‐Tau as well as Aβ (Figure 5E–K). Additionally, ELISA revealed a significant decrease in Aβ40 and Aβ42 levels in AAV9–shAGK mice (Figure 5L,M). Similarly, in SH‐SY5Y cells with AGK knockdown, we observed a significant decrease in tau protein levels and tau phosphorylation (Figure S5F–H), along with decreased levels of SQSTM1/p62 and increased levels of LC3B (Figure S5I,J), indicating enhanced autophagy. These results demonstrate that AGK downregulation promotes the clearance of tau pathology and soluble Aβ through autophagy‐mediated mechanisms [10]. Furthermore, cycloheximide chase experiments revealed that AGK inhibition accelerated tau protein degradation (Figure S5K–N), while qPCR showed that AGK knockdown in SH‐SY5Y cells did not alter tau mRNA levels (Figure S5O). These results suggest that AGK regulates tau primarily at the degradation level, likely through mitophagy‐mediated clearance. To further explore the effects of AGK downregulation on neurons, we examined the effects of AGK on neuronal morphology. AAV‐mediated AGK silencing in Aβ‐treated primary hippocampal neurons restored dendritic complexity, evidenced by increased total dendritic length (Figures 5N,O and S5P,Q) and Sholl intersection numbers (Figures 5P and S5R), indicating that AGK knockdown rescued neuronal morphology and neuroinflammation.

### 2.6 The Lipid Kinase Activity of AGK is Dispensable for Its Role in Mitophagy and Tau Pathology

To determine whether AGK's effects on mitophagy and Tau pathology require its lipid kinase activity, we generated a kinase‐dead AGK mutant (AGK G126E) and transfected N2A cells with control vector, AGK‐WT, or AGK G126E. Mitochondria–lysosome colocalization assays revealed that AGK‐WT overexpression significantly reduced colocalization under both basal and chloroquine (CQ)‐treated (30 µM, 24 h) conditions (Figure S6A,B), indicating that AGK‐WT suppresses mitophagy flux. However, the kinase‐dead G126E mutant did not alter colocalization compared with controls under either condition. Consistently, both AGK‐WT and G126E significantly increased Tau phosphorylation at Ser404, Ser396, and p62, and decreased the LC3‐II/LC3‐I ratio upon CQ treatment (Figure S6C,E–I). Lysophosphatidic acid (LPA) measurements confirmed that G126E abolished kinase activity without elevating LPA levels (Figure S6D). Collectively, these results demonstrate that AGK's lipid kinase activity is dispensable for its suppressive effects on mitophagy and its promotion of Tau pathology.

### 2.7 Downregulation of AGK Ameliorates Cognitive Dysfunction in APP/PS1 Mice

**FIGURE 6:**
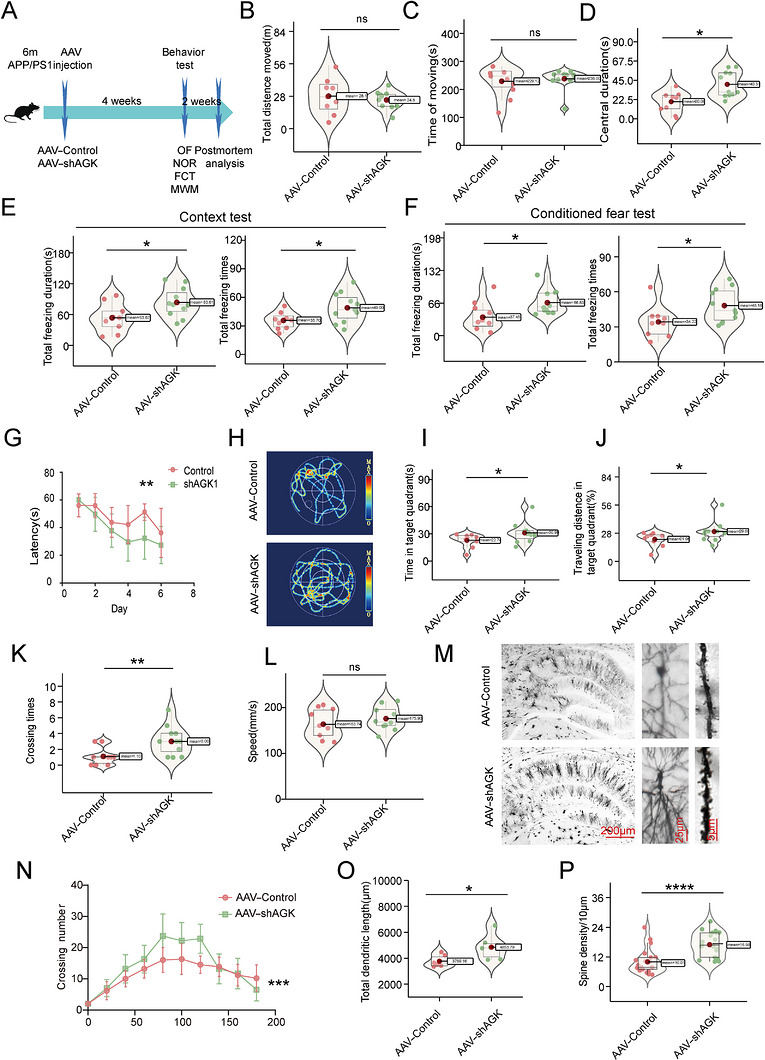
Downregulation of AGK improved synaptic and cognitive impairments in APP/PS1 mice. (A) Schematic diagram of AAV viruses’ injection and behavioral tests. *n* = 9, 11. (B and C) Total time (s) and total distance (m) of locomotion in the open field test. (D) Distance (m) traveled in the center zone during the open field test. (E) Total freezing time (left) and number of freezing episodes (right) in the contextual fear conditioning test. (F) Total freezing time (left) and number of freezing episodes (right) in the tone fear conditioning test. (G) Learning memory curve of mice in the MWM task (latency to find the hidden platform during the training period). (H) Movement trajectories of mice in the test 24 h after training. (I) Time spent in the target quadrant (s) by mice. (J) Percentage of distance traveled in the target quadrant by mice. (K) Number of platform crossings by mice. (L) Swimming speeds of the two groups of mice. (M) Representative dendrite from Golgi staining, scale bar: 200 µm. (N) Sholl analysis (*n* = 6 per group), (O) quantitative analyses of dendritic length (scale bar: 25 µm, *n* = 6 per group). (P) Averaged spine density (scale bar: 5 µm, *n* = 21 per group). *p  < 0.05, **p < 0.01, ****p < 0.0001 (Student's *t*‐test).

To determine if AGK downregulation improves cognition in AD, we injected APP/PS1 mice with AAV9–shAGK or control virus (Figure 6A). Behavioral tests revealed no altering general locomotion (Figure 6B,C). The open field test showed that AGK knockdown mice spent more time exploring the center zone (Figure 6D), indicating reduced anxiety or enhanced exploration. More importantly, in fear conditioning tests, the AAV9–shAGK group exhibited increased freezing time and frequency (Figure 6E,F), demonstrating enhanced contextual and cued fear memory. Spatial memory was assessed using the Morris water maze. During the probe trial, AGK knockdown mice spent more time and traveled a longer distance in the target quadrant (Figure 6H–J), with more platform zone entries (Figure 6K) and unchanged swimming speeds (Figure 6L), confirming improved spatial memory recall. At the structural level, Golgi staining revealed that AGK downregulation increased dendritic length and spine density (Figure 6M–P). This was supported by increased synaptic protein levels (Figure S7A–C) and increased Map2 immunoreactivity (Figure S7D,E), indicative of enhanced synaptic plasticity. Collectively, these data demonstrate that knocking down AGK rescues synaptic and cognitive deficits in APP/PS1 mice.

## 3 Discussion

Impairment of mitophagy is exacerbated in AD [33, 34, 35, 36]. Mitophagy dysfunction plays a crucial role in the pathogenesis and progression of AD [12], and the accumulation of dysfunctional mitochondria is a prominent hallmark of the disease [3]. The underlying mechanisms governing mitophagy impairment in AD remain incompletely understood, and effective therapeutic interventions are currently lacking. In our study, we have identified AGK as a key player in the mechanism of mitophagy impairment in AD, thereby providing a novel molecular target for therapeutic intervention. Specifically, we demonstrate that AGK upregulation in AD stabilizes the ATAD3A–TIM23 complex, facilitating PINK1 translocation to the inner mitochondrial membrane (IMM) and preventing its OMM accumulation. This process represents a previously unrecognized mechanism that contributes to mitophagy regulation in AD.

Notably, AGK knockdown reduced ATAD3A–TIM23 binding but did not affect ATAD3A–TOM40 interactions, suggesting selective disruption of the TIM23‐dependent import pathway. This aligns with evidence that PINK1 transport relies on membrane potential‐sensitive TIM23 complexes [37], while TOM40 likely participates in alternate transport routes. Previous research has shown that treatment with CCCP, a mitochondrial depolarization inducer, enhances the binding between PINK1 and the TOM complex [38]. However, it should be noted that since PINK1 is also transported through the TOM complex, promoting PINK1 transport could potentially increase the interaction between PINK1 and the TOM complex. Thus, the extent of interaction between PINK1 and the TOM complex during the process of promoting PINK1 transport may not be a meaningful indicator.

Although our study provides a potential avenue for understanding the inhibitory role of AGK in mitophagy by revealing its interaction with ATAD3A, several key limitations warrant consideration. First, we did not assess whether the lipid kinase activity of AGK directly modulates ATAD3A–TIM23 interactions. Future studies using kinase‐dead AGK mutants will be required to dissect its structural versus enzymatic roles. Second, the effects of AGK inhibition on mitophagy in glial cells remain to be investigated. Third, the potential involvement of AGK in intercellular mitochondrial transfer has not yet been explored. Furthermore, although previous studies have demonstrated that mitophagy suppresses Aβ and tau pathology and reverses cognitive deficits in AD models [12], how mitophagy alleviates these pathological features was not investigated in our study.

Neurons, as integral cells governing neural transmission within the human brain, exhibit a high metabolic demand [39, 40], which appears incongruous with their relatively sluggish mitophagy process. Despite the sluggishness of neuronal mitophagy, emerging evidence suggests that the clearance of damaged mitochondria in neurons can be facilitated through auxiliary mechanisms involving neighboring cells [41, 42]. Given that we only verify the effects of the AGK downregulation in enhancing mitophagy in neuron cells in vitro, it is conceivable that augmenting glial cell‐mediated mitophagy may potentially stimulate intercellular mitochondrial transfer between glial cells and neurons, thereby expediting the turnover and rejuvenation of neuronal mitochondria.

In addition, we investigated the impact of enhanced mitophagy on neuronal functions and Tau/Aβ pathology in AD. However, the precise mechanisms underlying these effects are not yet fully understood [10, 23]. We propose two possible explanations. First, the enhancement of mitophagy may help remove damaged mitochondrial components, reducing ROS levels that could alleviate the damage to the Tau clearance system. Second, mitophagy may decrease the number of damaged mitochondria and increase the proportion of healthy mitochondria that provide sufficient energy support for the neuronal Tau clearance system, such as lysosomes. As for the possible pathways, in AD, impaired mitophagy and mitochondrial dysfunction lead to reduced ATP production, inducing AMP‐activated protein kinase (AMPK) activation (p‐AMPK) that induces tau phosphorylation, a key mediator of the synaptotoxicity of Aβ1–42 oligomers, suggesting a self‐reinforcing loop that amplifies p‐Tau pathology.

Finally, AGK represents a promising therapeutic target for AD: by promoting PINK1 accumulation (mitophagy initiation) rather than later Parkin‐dependent steps, AGK inhibition may bypass Parkin dysfunction observed in late‐stage AD. While no AGK‐specific inhibitors exist. Notably, AGK's homology to other lipid kinases (e.g., diacylglycerol kinases) may enable repurpose of existing kinase modulators.

In conclusion, our overall research findings indicate that AGK acts as a scaffolding factor that stabilizes the TOM–TIM supercomplex, which in turn prevents PINK1 accumulation on the OMM and suppresses mitophagy. In the context of AD, downregulation of AGK alleviates mitophagy deficiency, thereby ameliorating mitochondrial dysfunction and disease pathology. These findings provide novel insights into the molecular mechanisms underlying mitophagy and highlight AGK as a potential therapeutic target for AD.

## 4 Material and Methods

### 4.1 Antibodies

The details of the primary and secondary antibodies used in this study are shown in Table S1.

### 4.2 Animal Models and Housing Conditions

Pregnant C57BL/6J were sourced from the Experimental Animal Center at Tongji Medical College, Huazhong University of Science and Technology. Male C57BL/6J and APP/PS1 mice were came from Charles River Laboratories [43] and Male 3×TG mice were available through the JAX MMRRC (Stock# 034830) [44]. At 6 months of age, APP/PS1 mice were subjected to viral injection, followed by behavioral experiments after a 1‐month to evaluate cognitive function. Subsequently, at 7 months of age, mice were euthanized for molecular biology and biochemical analyses. The AD mouse model used 6‐month‐old APP/PS1 mice. The mice were randomly divided into AAV9–control and AAV9–shAGK groups.

### 4.3 Human Brain Tissue Specimens

Postmortem human brain samples were obtained by dissecting frozen brains from five cases diagnosed with AD (age 82 ± 10.2 years, mean ± SD) and six nondemented controls (age 80 ± 11.1 years) sourced from the Brain Bank of China (Zhejiang University School of Medicine). AD diagnosis was made based on the criteria outlined by the Consortium to Establish a Registry for AD and the National Institute on Aging, confirmed through the identification of amyloid plaques and neurofibrillary tangles in formalin‐fixed tissue. The post‐mortem interval was comparable between the AD group and the control group. Additional comprehensive details can be referenced in Table S2.

### 4.4 Primary Neuron and Cell Line Culture

The HEK‐293T (ATCC CRL‐11268), SH‐SY5Y (ATCC CRL‐2266), and N2a (ATCC CCL‐131) cell lines used in this study were obtained from ATCC. HEK293/T cells were maintained in a culture medium composed of Dulbecco's modified eagle medium (DMEM) supplemented with high glucose concentration, specifically DMEM‐high glucose, and supplemented with 200 mg/mL G418, along with 10% FBS (fetal bovine serum). On the other hand, SH‐SY5Y and N2A cells were cultured in regular DMEM‐high glucose medium supplemented with 10% FBS. All cell lines were cultured at 37°C in a humidified atmosphere containing 5% CO_2_. All cell lines used in this study (SH‐SY5Y, HEK293T, N2A) have undergone authentication by short tandem repeat analysis to confirm their identity and tested mycoplasma free.

For the mouse primary neuron, the plating medium (DMEM/F12 supplemented with 10% FBS) and the maintenance medium (neurobasal medium supplemented with 1× B27, 1×GlutaMAX, and 100 U/mL penicillin–streptomycin) were freshly prepared. Then, the pregnant mouse was anesthetized, the fetus was removed, and each fetus was washed with saline and 75% ethanol before placing them on ice. Forceps were used to remove the mouse brain and separate the hippocampus on the filter paper. The isolated hippocampus was transferred to the glass bottle containing 2 mL of D‐Hanks solution. Ophthalmic scissors were used to cut the hippocampus. After cutting, 2 mL of 0.125% trypsin was used for digestion. A 40 µm cell strainer was placed on top of a 50 mL centrifuge tube and the digested cells were filtered. A certain amount of planting medium was added to the cell culture plate (usually 1 mL for a six‐well plate, 800 µL for a 12‐well plate). After seeding, the cells were placed in the incubator and culture them. After 4 h, the planting medium was replaced with maintenance medium, within a maximum of 6 h. For a six‐well plate, the medium was changed with maintenance medium every 3 days, and for a 12‐well plate, the medium was changed every 4 days (partial medium change). Mouse primary neurons were isolated from mouse embryos (embryonic Days 16–19), which were cultured in a CO_2_ incubator at 37°C. Aβ_1–42_ polypeptide was preserved in freeze‐dried powder at −80°C. When used, the powder was dissolved in dimethyl sulfoxide (DMSO), diluted to 0.2 mM with DMEM medium, overnight at 4°C, to prepare Aβ oligomers. Primary cells were treated with 2 µM Aβ oligomers at 37°C for 48 h.

### 4.5 Oxygen Glucose Deprivation

The cell culture medium was removed and the cells were rinsed several times with glucose‐free DMEM. The cells were cultured in glucose‐free DMEM for 6 h under the following conditions: 37°C, 0.5% O_2_, 94.5% N_2_, and 5% CO_2_.

### 4.6 Viral Transduction

Virus packaging was performed using psPAX2, PLKO.1 (shRNA/shMock), and PMD2G. Both the infected and uninfected SH‐SY5Y cells were selected using a culture medium containing 4 µg/mL puromycin (N2A cells were selected using a culture medium containing 2 µg/mL puromycin). When all cells in the negative control group had died, the infected cells were further cultured for 1 more day.

### 4.7 Protein Extraction for Tau and Aβ Detection

For the analysis of Tau, p‐Tau, mouse hippocampal tissues were homogenized using a motorized pestle in RIPA lysis buffer supplemented with 1% SDS, 1 mM PMSF, and a complete protease and phosphatase inhibitor cocktail. The use of high‐concentration SDS in the lysis buffer was critical for the efficient solubilization of aggregated and hyperphosphorylated Tau species. The homogenate was incubated on ice for 30 min and then sonicated on ice using a probe sonicator at 30% amplitude for three cycles of 5 s each, with 10‐s intervals between cycles. Subsequently, the lysate was centrifuged at 12,000×*g* for 15 min.

For the analysis of Aβ protein levels, mouse hippocampal tissues were homogenized in ice‐cold RIPA buffer (50 mM Tris–HCl, pH 7.4, 150 mM NaCl, 1% NP‐40, 0.5% sodium deoxycholate, 0.1% SDS) containing protease inhibitor cocktail. Homogenates were centrifuged at 100,000×*g* for 1 h at 4°C. The supernatant was collected as the RIPA‐soluble fraction. The remaining pellet was washed once with RIPA buffer and then resuspended in 5 M guanidine–HCl in 50 mM Tris–HCl (pH 8.0) supplemented with protease inhibitors. Following incubation, the RIPA‐insoluble fraction was obtained by centrifuging the homogenate at 20,000× *g* for 15 min at 4°C. The levels of human Aβ40 and Aβ42 were quantified using ELISA kits

### 4.8 Coimmunoprecipitation and Mass Spectrometry Analysis

SH‐SY5Y cells were seeded in a 10 cm culture dish. Upon reaching confluency, the cells were lysed using IP lysis buffer and subjected to ultrasonication at 5% power. After centrifuging, the supernatant was collected in a new 1.5 mL EP tube and adjusted for concentration. 60 µL of the supernatant was mixed with 20 µL of 4×loading buffer to prepare the sample. In the remaining supernatant, 20 µL of agarose A/G and 0.25 µL of IgG (matching the subsequent target antibody) were added and incubated at 4°C for 1–3 h. The mixture was then centrifuged at 3000×*g* for 3 min. The collected supernatant was combined with 6 µg of the target antibody and 20 µL of protein agarose A/G. The mixture was incubated with rotation at 4°C for 24 h. After centrifuging, the immunocomplexes were washed with phosphate‐buffered saline (PBS), repeating the washing process six times.

AGK proximity interactome by IP according to the following steps: HEK293T cells were transfected with pCMV–AGK–Flag for 48 h and harvested for IP using Flag antibody. Protein samples were collected in‐gel about 1 cm × 1 cm in size and analyzed by LC–MS.

### 4.9 IF and Image Analysis

SH‐SY5Y cells from the control group and AGK knockdown group were washed three times with PBS for 4 min. Subsequently, the cells were fixed with 4% paraformaldehyde for 15–20 min, followed by three additional washes with PBS. To facilitate membrane permeabilization, the cells were treated with 0.5% Triton X‐100 in PBS for 20–40 min and then washed three times with PBS. Blocking was performed at room temperature for 0.5–1 h using 3% bovine serum albumin (BSA) solution in PBS, without sodium azide. After removing the blocking solution, the cells were incubated with appropriately diluted TIM23 antibody (1:200) at 4°C for 16 h. Following the incubation period, the cells were washed three times with PBS and subsequently incubated with appropriately diluted LAMP2A antibody (1:500) for 16 h. The cells were then washed three times with 1× PBS. Corresponding fluorescent secondary antibodies were applied and allowed to incubate at room temperature for 1 h. Finally, the cells were washed three times with 0.1% Triton X‐100 in PBS, mounted on slides, and fluorescence images were captured. All fluorescence images were captured using a Zeiss LSM 800 laser scanning confocal microscope (Zeiss, Jena, Germany). The colocalization of TIM23 and LAMP2A was quantified using the Coloc 2 plugin in ImageJ.

Brain slices were fixed with 4% paraformaldehyde for 4 min, followed by 0.5% Triton X‐100 for 0.5 h and blocked using 3% BSA for 1 h. They were subsequently incubated with the primary antibody overnight at 4°C, washed three times by PBS for 10 min each time, followed by secondary antibodies at 37°C for 1 h. Slice Scanning System (VS200; Olympus, Japan). ImageJ software was used for image capture. The Iba1+ microgliosis density and GFAP+ astrogliosis density was analyzed using ImageJ software. Colocalization was quantified using the Coloc 2 plugin in ImageJ.

### 4.10 Stereotaxic Injection

The AD mouse model used 6‐month‐old APP/PS1 mice. The mice were randomly divided into AAV9–control and AAV9–shAGK groups. Under continuous low‐dose isoflurane anesthesia, stereotaxic injections were performed into the hippocampus of the mice (anteroposterior, −2.18 mm; mediolateral, ±1.5 mm; depth, −1.5 to −2.0 mm from bregma). Before injection, the electrode needle descends to a depth of −2.0 and then rises back to a depth of −1.5 before the injection is performed. AAV9–control and AAV9–shAGK viruses were injected at a rate of 0.2 µL/min using a Hamilton syringe, with 2 µL of high‐titer AAV9 (∼10^12^ GU/mL) injected into each side. After completion of the injections, the needle was slowly withdrawn after 2 min. The incision was sutured, and a combination of neomycin and lidocaine gel was applied. Behavioral experiments were conducted 1 month later, and tissue samples were collected 2 week after the experiments for protein immunoblotting and Golgi staining.

### 4.11 Behavioral Assessments

The details of the behavior experiments used in this study are shown in Supporting Information.

### 4.12 Golgi Staining

The Golgi–Cox was prepared solution by mixing 5% potassium dichromate, 5% mercuric chloride, 5% potassium chromate, and water in a ratio of 5:5:4:10. The solution was allowed to sit in a dark place for 5 days. The mouse brain was removed and placed in the prepared Golgi–Cox solution, allowing it to sit in the dark for 14 days, changing the fresh Golgi–Cox solution every 2 days. After 2 weeks of immersion, the brain was transferred to a 30% sucrose solution, changing the solution every 2 days until the brain settles, proceeding to vibratome sectioning. The blade was soaked in xylene for 5 min in a fume hood and dried for use in vibratome sectioning. 6% sucrose was poured into the vibratome chamber (to increase tissue block toughness and reduce fragmentation) and the brain was sliced at a thickness of 80 µm. The sections were mounted and were air dried before placing them in a humidified chamber. Then, Golgi staining was performed: CXA solution was prepared in a fume hood by mixing equal amounts of chloroform, xylene, and absolute ethanol. Under dark conditions, the brain sections were immersed sequentially in water for 1 min, ammonia for 50 min, and double‐distilled water for 1 min. Then, an ethanol gradient dehydration was performed, followed by a 15‐min immersion in CXA solution, followed by neutralizing with a neutral mounting medium and the slides were then air dried. The number of branches were analyzed and quantified using ImageJ.

### 4.13 Isolation and Purification of Mitochondria

To isolate mitochondria from hippocampal tissue for protein immunoblotting experiments, the following steps were performed. Approximately 50–100 mg of tissue was dissected and then finely minced on ice after washing with PBS. The tissue was homogenized with ten times the volume of tissue in prechilled mitochondrial isolation reagent A containing PMSF. Homogenization was performed evenly for 10 strokes at 1000×*g*, 4°C, followed by centrifugation for 5 min. The supernatant was carefully collected and subjected to further centrifugation at 3500×*g*, 4°C, for 10 min. The resulting pellet represented the mitochondrial protein fraction, while the supernatant contained the cytoplasmic proteins. The pellet was resuspended in lysis buffer for subsequent protein analysis using the BCA method to determine protein concentration.

### 4.14 Assessment of Mitochondrial Membrane Potential

The cells were seeded into individual wells of a six‐well plate and allowed to reach confluence. Subsequently, the cells were stained using 50 nM MitoTracker Green FM and 150 nM MitoTracker Red CMXRos, following incubation at 37°C for 15 min. Flow cytometry was then employed to facilitate quantitative analysis of the stained cells. For the preparation of MitoTracker Red CMXRos, the dry powder was dissolved in 93 µL of DMSO to obtain a stock solution with a concentration of 1 mM. Prior to staining, a working solution with a final concentration of 150 nM was prepared by diluting the stock solution in a serum‐free culture medium. Following the completion of the staining procedure, the cells were detached, collected, and subsequently analyzed using a flow cytometer. Moreover, to visualize the mitochondria labeled with the aforementioned dyes, confocal microscopy was employed, and the acquired images were subjected to analysis using ImageJ software. MitoTracker Green FM, which exhibits fluorescence independent of mitochondrial membrane potential, emitted green fluorescence upon binding to mitochondria, serving as a marker for total mitochondrial. Conversely, MitoTracker Red CMXRos, whose fluorescence intensity is positively correlated with mitochondrial membrane potential, displayed red fluorescence upon binding to mitochondria, enabling assessment of mitochondrial membrane potential.

### 4.15 Transmission Electron Microscopy

The injected viral brain region was dissected into small cubes of approximately 1 cubic millimeter. The tissue was fixed in a 0.1 M carbonate‐buffered solution containing 3% glutaraldehyde and 2% paraformaldehyde (pH 7.3) and then subjected to a 0.1 M sodium carbonate wash. Subsequently, the samples were treated with 0.1% tannic acid followed by fixation with 1% osmium tetroxide for 30 min. Finally, staining was performed using 1% uranyl acetate. Following dehydration, the samples were embedded in LX‐112 and polymerized for 2 days at 60°C. Postpolymerization, the samples were sectioned using an ultracut microtome (Leica) and imaged using a high‐resolution transmission electron microscope.

### 4.16 Molecular Docking

Molecular docking between TIM23 and ATAD3A was performed using ClusPro. The crystal structure of AGK (PDB ID: 7CGP) and ATAD3A (AlphaFoldDB ID: AF‐Q9NVI7‐F1) were obtained from protein data bank (http://www.rcsb.org) and AlphaFold Protein Structure Database (https://alphafold.ebi.ac.uk). The lowest‐energy docking solutions from the top 10 search results were chosen.

### 4.17 Datasets Collection and Processing

Age‐ and AD‐related datasets (GSE272485, GSE33000) were retrieved from the Gene Expression Omnibus (GEO) database. Raw data were normalized using R packages (“limma” for GSE33000; “DESeq2” for GSE272485) to correct batch effects and adjust for technical variability. For GSE272485, AGK expression‐age relationships were evaluated using Pearson correlation and “WGCNA” in R. WGCNA was used to construct coexpression modules of highly correlated genes and analyze their correlation with age [45]. For GSE33000, control samples were selected for age‐related analysis. Differential expression genes were identified using the “limma” R package with criteria set at *p* < 0.05, and rows (genes) of the GSE33000 data with valid signals (>−0.1) in at least 75% of samples were retained for downstream analysis. Missing values (NA) were excluded during the filtering process to avoid biased removal of biologically relevant features. Mitochondrial function‐related gene sets were obtained from Genecard.

### 4.18 Statistical Analysis

Data were expressed as mean ± SD and analyzed using GraphPad statistical software. Prior to statistical analysis, appropriate methods were selected based on the number of groups being compared. For two‐group comparisons, normality of the data and equality of variances were assessed using normality tests and F‐tests, respectively. These tests aimed to determine whether the data followed a normal distribution and exhibited homogeneity of variance. Subsequently, based on the results obtained from these tests, either a nonpaired *t*‐test (Student's *t*‐test) or Mann–Whitney *U* test was employed to evaluate statistical differences between the groups. In the case of multiple groups, the one‐way ANOVA was conducted if the data satisfied the assumptions of normality and homogeneity of variance. Statistical significance was assessed at *p* < 0.05.

## Author Contributions

XW designed and oversaw the research project and directed most of the experiments, analyses, and writing. QD, JW, and GW planned and organized most of the experiments, analyzed, and interpreted the data. WL, XY, and CG planned and performed all experiments. YH, ZW, and YL assisted with the stereotaxic injection of virus and behavioral studies and analyses. JW, WJ, and RL proofread our manuscript. All authors have read and approved the final manuscript.

## Funding

This work was supported in parts by grants from National Natural Science Foundation of China (82330041 and 82571372) and grant from Science and Technology Innovation Team project to Xiaochuan Wang from Department of Science and Technology of Hubei Province (2022‐72‐18).

## Ethics Statement

The postmortem human brain tissues used in this study were sourced from the Zhejiang Brain Bank, which had obtained written informed consent for tissue donation and research use from donors or their legal representatives in accordance with institutional guidelines and ethical regulations. All experimental protocols involving human postmortem brain samples were approved by the Medical Ethics Committee of Zhejiang University School of Medicine (Approval Number: 2020‐005). All animal experiments were approved by the Animal Care and Use Committee of Huazhong University of Science and Technology (IACUC Approval Number: 4942). All procedures were performed in strict accordance with the National Institutes of Health Guide for the Care and Use of Laboratory Animals.

## Consent

All authors consented to the publication of this paper.

## Conflicts of Interest

The authors declare no conflicts of interest.

## Supporting information




**Supporting Information**: mco270863‐Sup‐0001‐SuppMatt.docx
Additional supporting information can be found online in the Supporting Information section.

## Data Availability

The datasets used and/or analyzed during the present study are available from GEO (https://www.ncbi.nlm.nih.gov/geo/)
