# Supplementary material for: Acylglycerol Kinase Inhibition Restores Mitophagy and Alleviates Alzheimer's Disease Pathology
Source: MedComm (2020). 2026 Jul 8;7(7):e70863. doi: 10.1002/mco2.70863 (PMC13346768; doi:10.1002/mco2.70863)
Supplement: Supplementary file 1 — Supporting Information: mco270863‐Sup‐0001‐SuppMatt.docx Additional supporting information can be found online in the Supporting Information section. [file MCO2-7-e70863-s001.docx]

**Supplementary Materials for**

**AGK Inhibition Restores Mitophagy and Alleviates Alzheimer’s Disease Pathology**

Wensheng Li#^1,2^, Xuan Yu#^3^, Cuiping Guo#^3^, Yuting Huang^4^, Zhen Wei^5^, Yi Liu^3^, Jian-Zhi Wang^2,3,6^, Rong Liu^3^, Weike Ji^1^, Qiuhong Duan^*1^, Jing Wang^*7^, Guihua Wang^*4^, and Xiaochuan Wang^*2,3,6,8^

^1^Department of Biochemistry and Molecular Biology, School of Basic Medicine, Tongji Medical College, Huazhong University of Science and Technology, Wuhan, 430030, Hubei, China.

^2^Co-innovation Center of Neurodegeneration, Nantong University, Nantong 226001, China.

^3^Department of Pathophysiology, School of Basic Medicine, Tongji Medical College, Huazhong University of Science and Technology, Wuhan, 430030, China.

^4^GI Cancer Research Institute, Tongji Hospital, Huazhong University of Science and Technology, Wuhan, 430030, China.

^5^Department of pathology, Maternal and Child Health Hospital of Hubei Province, Tongji Medical College, Huazhong University of Science and Technology, Wuhan, 430030, China.

^6^Institutes of Biomedical Sciences, School of Medicine, Hubei Key Laboratory of Cognitive and Affective Disorders, Jianghan University, Wuhan 430056, China

^7^Department of Immunology School of Basic Medicine, Tongji Medical College, Huazhong University of Science and Technology, Wuhan, 430030, China.

^8^Lead contact

#The authors contributed equally to this work.

^*^Correspondence: [wxch@mails.tjmu.edu.cn](mailto:wxch@mails.tjmu.edu.cn) (X.W.), [ghwang@tjh.tjmu.edu.cn](mailto:ghwang@tjh.tjmu.edu.cn) (G.W.), [wangjhxh@163.com](mailto:wangjhxh@163.com) (J.W.), [duanqhwz@hust.edu.cn](mailto:duanqhwz@hust.edu.cn) (Q.D.)

**The file includes:**

Materials and methods

Figure S1 to S7

Table S1 to S3

##### Material and methods

**Reagents**

MitoTracker Green FM (Thermo Fisher Scientific, M46750, USA); MitoTracker™ Red CMXRos (Thermo Fisher Scientific, M7512, USA); Protein A/G PLUS-Agarose (SantaCruz Biotechnolog, sc-2003, USA); T4 DNA Ligase Buffer (Thermo FisherScientific, B69, USA); β-Amyloid_1-42_ (Chinapeptides, 04010011526, China); ™Neurobasal (Thermo Fisher Scientific, 21103049, USA); 1×B27(Thermo Fisher Scientific, 17504044, USA); Anti-fluorescence quenching sealing solution (BeyotimeBiotechnology, P0131, China); Phenylmethanesulfonyl fluoride (Thermo Fisher Scientific,26616, USA); Tissue mitochondrial isolation kit (BeyotimeBiotechnology, C3606, China); Pancreatic enzyme cell digestive fluid (BeyotimeBiotechnology, C0201, China); Penicillin-streptomycin (Thermo Fisher Scientific, 15140122, USA); Lipofectamine™ 2000 (Thermo Fisher Scientific, 11668019, USA); BCA Protein Assay Kit (Beyotime Biotechnology, P0012, China), Human Amyloid beta 42 ELISA Kit(Thermo Fisher Scientific, KHB3441, USA), Human Amyloid beta 40 ELISA Kit(Thermo Fisher Scientific, KHB3481, USA),CHX(MCE, 66-81-9, USA). RIPA lysis buffer (Beyotime Biotechnology, P0013B); SDS (Sodium Dodecyl Sulfate, Sigma-Aldrich, L3771); PMSF (Beyotime, ST506); protease and phosphatase inhibitor cocktail (Yeasen, 20124ES03); Human lysophosphatidic acid (LPA) Elisa kit (CUSABIO, CSB-EQ028005HU).

**Plasmids and viruses**

Plasmids: psPAX2, PLKO.1-shMock, PMD2.G purchased from Addgene (USA). pCMV-AGK-Flag, pCMV-AGK-HA plasmids were obtained from our own laboratory. AAV9-AGK, AAV9-shAGK virus was constructed and packaged by Genechem (China). The siRNA target sequence of AAV9-shAGK virus is 5′-*CGCTGAGTACTTCGAAATGTC*-3′.

To silence AGK expression, Lentivirus was employed in SH-SY5Y and N2A (PARKIN overexpression) cell lines. For AGK knockdown in SH-SY5Y cells, two distinct shRNA sequences, AGKia (5′-*CATCAAGCCTCTATCTCATAC*-3′) and AGKib (5′-*GCCCTTCCATTTCTCTTCTTT*-3′), were utilized. And then, a non-targeting control sequence (5′-*CGCTGAGTACTTCGAAATGTC*-3′) was employed to assess the specificity of the knockdown effect. In N2A cells, the same shRNA sequence (5′-*CGCTGAGTACTTCGAAATGTC*-3′) was employed to silence AGK expression.

**Quantitative real-time PCR**

Samples were subjected to total DNA extraction using the TIANamp Genomic DNA Kit. 10 ng DNA was isolated from each sample, and qPCR was performed to determine the copy number of mitochondrial DNA (mtDNA). The mtDNA quantity was normalized to nuclear DNA (nDNA) as an internal control. Primers were designed as shown in Table S3, and Quantitative polymerase chain reaction (PCR) was performed in a 20 μl standard PCR reaction mixture by the manufacturer’s protocol (Hifair® III One Step RT-qPCR SYBR Green Kit, Yeasen Biotech, 11143ES50, Shanghai, China).

**Western blot (WB)**

Cell and brain tissue samples were lysed using either weak or strong RIPA buffer and subjected to sonication. The lysates were then centrifuged at 4 °C, and the supernatant was collected for protein quantification using the BCA kit with 5 μL of the sample. The remaining samples were supplemented with 4x loading buffer and heated at 95 °C for 10 minutes. The extracted proteins were resolved by SDS–polyacrylamide gel electrophoresis and then transferred onto nitrocellulose membranes (Amersham Biosciences, USA). The membrane was blocked with 5% skim milk for 50 minutes and then incubated with the primary antibody at 4 °C for 15 hours. On the following day, the membrane was washed with TBST and incubated with the secondary antibody at room temperature for 1 hour. After washing the membrane with TBST, band intensities were visualized using the Odyssey infrared imaging system (LICOR Biosciences, USA). Finally, the band intensity readings were obtained using ImageJ (Fiji) software.

**Open-field (OF)**

The mice were arranged in an empty area for 5 min. The time and distance of moving in central region were recorded.

**Fear conditioning test (FCT)**

The experiment was conducted in a white box (33cm × 33cm × 33cm) equipped with a transparent front door, grid floor, and a speaker. On the first day, mice were placed in the box for a 3-minute acclimation period, followed by a 20 s, 80 dB, 2,000 Hz auditory stimulus, immediately followed by a 2 s, 0.8 mA foot shock. After a 60 s interval, this sequence was repeated three times. The contextual fear test was conducted 24 h later. Mice were placed back into the same box without any auditory stimulus, and their freezing time and freezing episodes were recorded for 5 min. On the third day, the auditory fear conditioning test was performed. Mice were placed in a box with different background cues, including walls of different colors and a smooth plastic floor. After 2 minutes of free exploration, a 20 s, 80 dB, 2,000 Hz auditory stimulus was presented, and freezing time and freezing episodes were recorded in the various environments for a period of 5 min.

**Morris water maze test (MWM)**

The detection of spatial learning and memory was performed using the Morris water maze (MWM) paradigm. The experiment was conducted in a circular water tank with a diameter of 1.2 m and a height of 50 cm, divided into four quadrants. The water in the tank was supplemented with non-toxic white dye. The experiment was tested over on sixth days, with mice undergoing consecutive training for five days. Each day, the mice were subjected to three trials, where they were placed into the tank from different quadrants and allowed to swim for 1 min. After 24 h, the platform was removed, and the mice were allowed to freely swim in the pool for 60 seconds. Various parameters related to the mice's movement were recorded.

**Novel objection recognition test (NOR)**

On the initial day of the experiment, mice were placed into a square container without a lid, starting from the same designated position. The container contained two distinct objects, denoted A and B, characterized by their varying shapes and colors. The mice were provided 5 min to familiarize themselves with objects A and B, during which their interactions with the objects were meticulously observed and recorded. Following a 2-hour interval, object A was replaced with a dissimilarly shaped and differently colored object, designated as C. The aforementioned procedure was repeated, and the mice's interactions with objects C and B were meticulously recorded. Subsequently, after a lapse of 24 h, object C was substituted with yet another object, denoted as D, and the mice's interactions with objects D and B were once again recorded. The recorded parameters encompassed the frequency of nose contacts with the objects and the duration of exploratory behavior exhibited towards the objects. The recognition index was computed as follows: TA/(TA+TB) for the initial day, TC/(TC+TB) after 2 h, and TD/(TD+TB) after 24 h. Here, TA, TB, TC, and TD represent the cumulative exploration time devoted to objects A, B, C, and D, respectively.

##### Supplementary figure legends


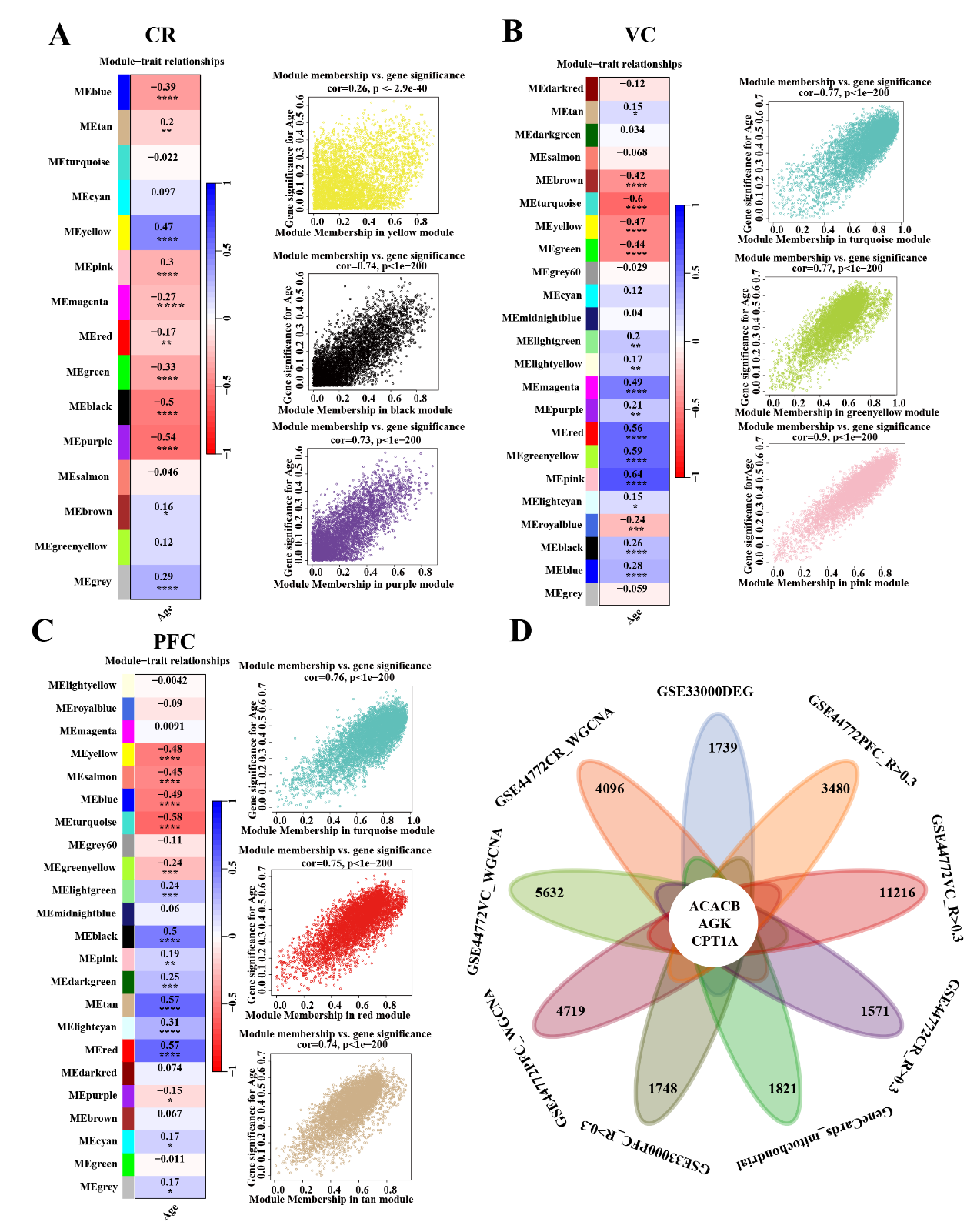


**Figure S1.** Identification of age-related and upregulated mitochondrial function genes in AD

**(A)**, **(B)**, **(C)** WGCNA was used to construct co-expression modules in three brain regions (CR, VC, PFC) and analyze their correlation with age. The right panels show scatter plots of module membership versus gene significance for the top three modules with the strongest correlations, displaying the correlation coefficient (cor) and p-value. **(D)** The petals in different colors represent different dataset processing steps. GSE47721CR_WGCNA, GSE47721VC_WGCNA, and GSE47721PFC_WGCNA represent gene modules obtained from (A), (B), and (C), respectively. GSE47721CR_R > 0.3, GSE47721VC_R > 0.3, and GSE47721PFC_R > 0.3 represent genes directly obtained from Pearson correlation analysis with age (R > 0.3) in the three brain regions. GeneCards_mitochondrial represents mitochondrial function-related genes obtained from GeneCards. GSE33000DEG represents differentially expressed genes obtained from the GSE33000 dataset. Finally, three genes were identified: AGK, CACB, and CPT1A.


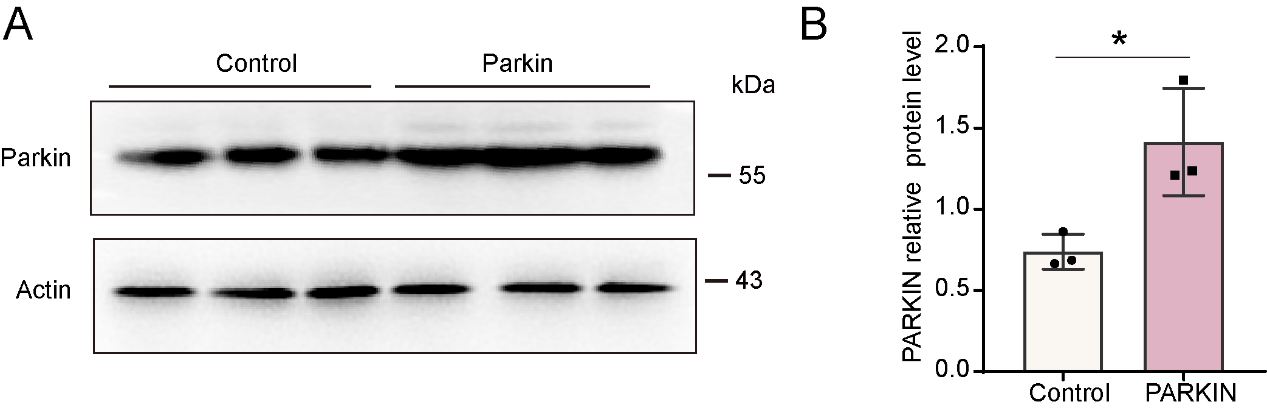


**Figure S2.** Establishment of a PARKIN -overexpression stable cell line.

(**A**) Stable SH-SY5Y cell lines with PARKIN overexpression were established. Protein levels of PARKIN were detected using WB. (**B**) Statistical graphs representing the protein levels of PARKIN, normalized to Actin. The graph reports mean±SD, n=3, *: P < 0.05.


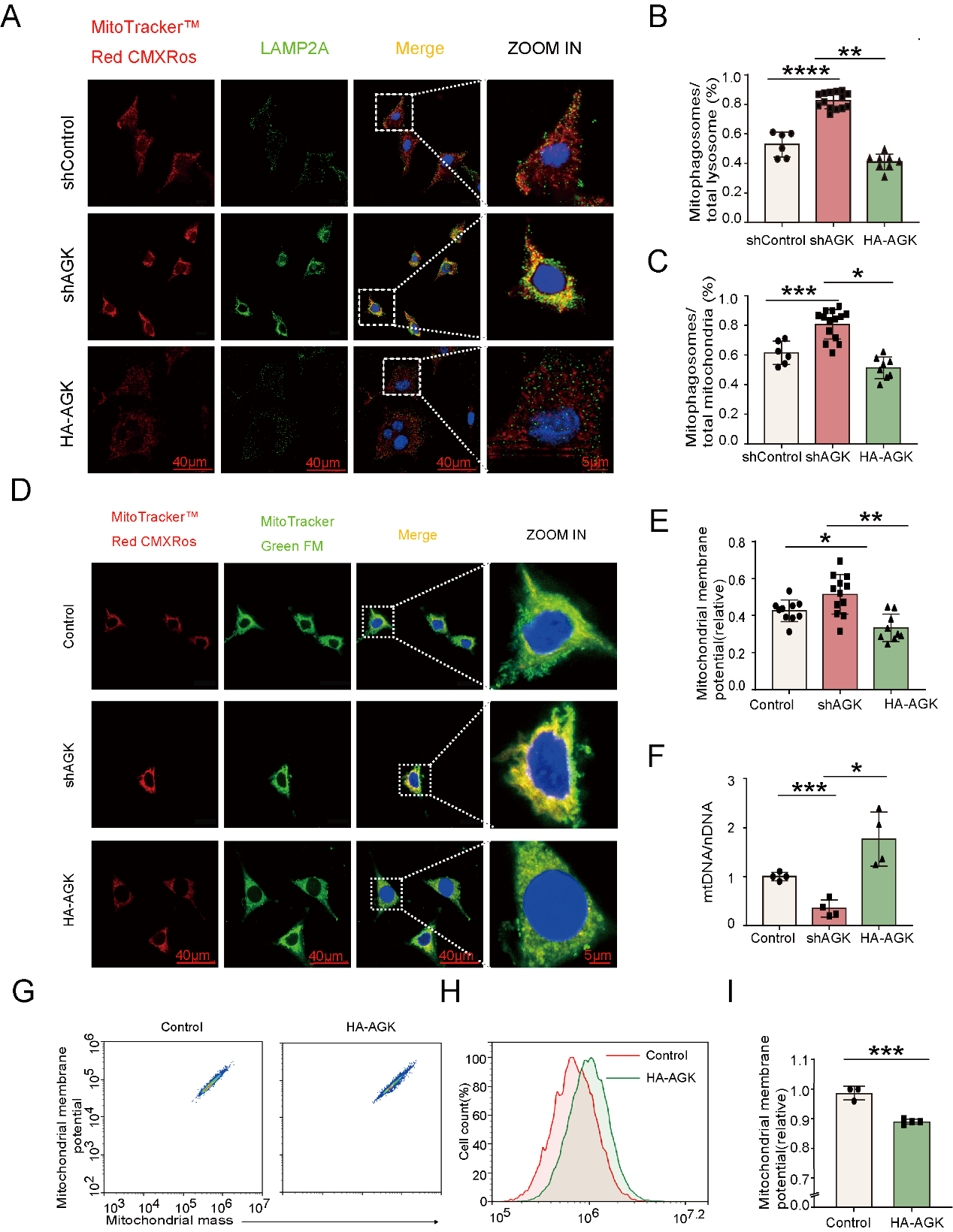


**Figure S3.** Downregulation of AGK reduced damage to mitochondria in SH-SY5Y

(**A**) Confocal microscopy images of SH-SY5Y cells in Control, AGK silenced(shAGK), and HA-AGK groups with MitoTracker™ Red CMXRos labeling mitochondria (red) and LAMP2A antibody labeling lysosomes (green). Scale bar, 40 μm and 5 μm (zoom in). (**B and C**) Frequency of colocalization of mitochondria and lysosomes, normalized to total mitochondria or lysosomes respectively, n=6,14,8 (**D**) Representative images of immunostainings showed co-stained with MitoTracker Green FM and MitoTracker™ Red CMXRos in Control, shAGK, and HA-AGK groups. Scale bar, 40 μm and 5 μm (zoom in). (**E**) Frequency of colocalization of red with green fluorescence (relative mitochondrial membrane potential) in Control, shAGK, and HA-AGK groups, as in (**D**), n=10,12,9. (**F**) Statistical analysis of relative mtDNA content in Control, shAGK, and HA-AGK groups, normalized to nDNA as a reference, n=4. (**G**) Flow cytometry analysis of live cells co-stained with the two aforementioned dyes in Control and HA-AGK groups. (**H**) Red fluorescence peak (membrane potential) in flow cytometry analysis of Control and HA-AGK groups. (**I**) Statistical analysis of the average ratio between red and green fluorescence intensities, representing relative membrane potential as in (**G**), n=3,4. * P < 0.05, ** P < 0.01, *** P < 0.001, **** P < 0.0001(Student’s t-test).


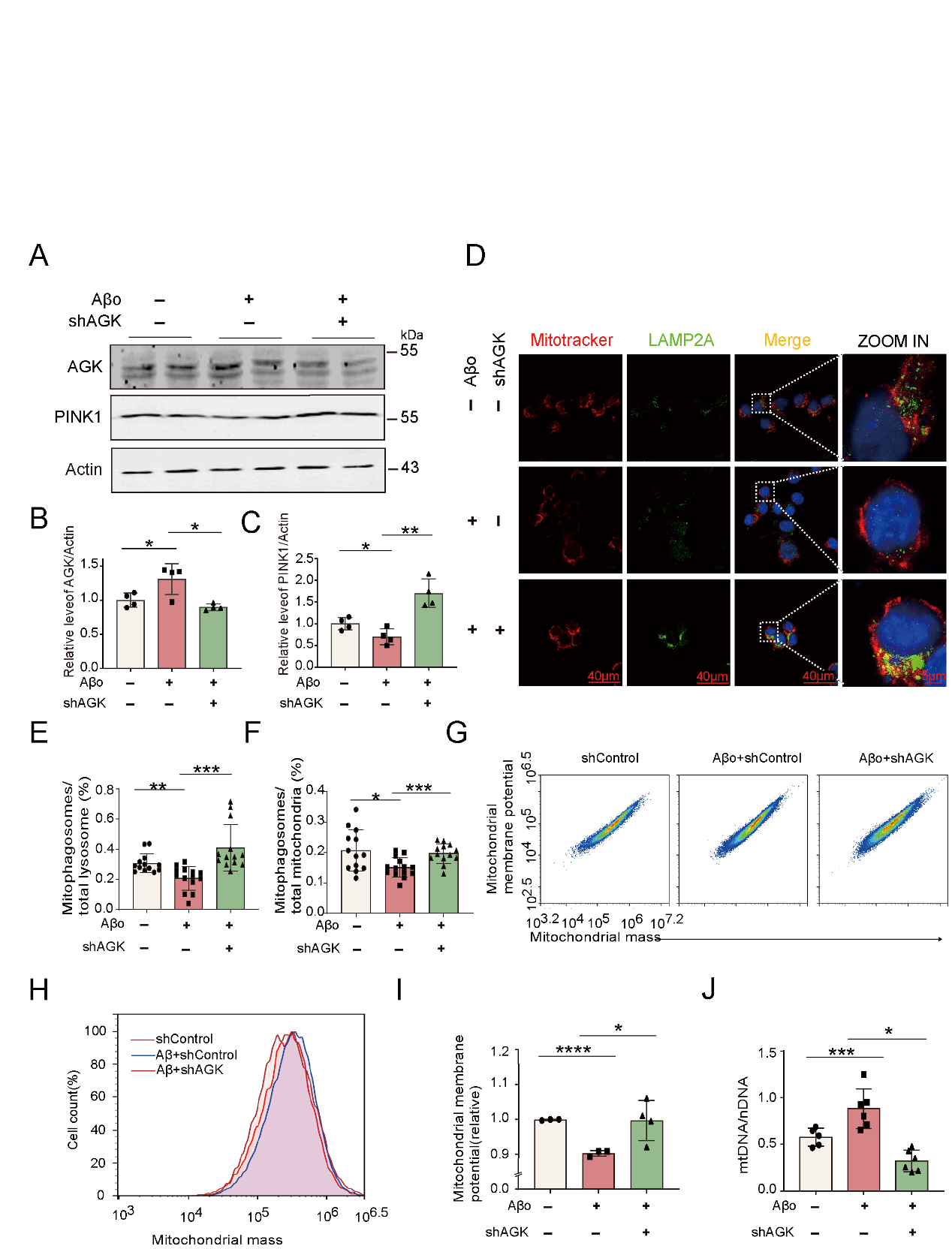


**Figure S4.** AGK deficiency blocked Abeta-induced mitochondrial dysfunction in N2A cells

(**A**) Total protein from N2A cells of shCTR, Aβo+shCTR and Aβo+shAGK groups were analyzed by immunoblotting. (**B**) Statistical analysis of AGK protein levels (n=4 in each group). (**C**) Statistical analysis of PINK1 protein levels (n=4 in each group). (**D**) Co-staining of MitoTracker Green FM and MitoTracker™ Red CMXRos in N2A cells from the Control group, as well as in N2A cells from the Control group and AGK silenced group after Aβ treatment, observed under confocal microscopy. Scale bar represents 40 μm and 5 μm (zoom in). (**E and F**) Quantification of the red fluorescence to green fluorescence ratio in the aforementioned three groups, representing relative membrane potential, n=13,14,13. (**G**) Collection of live cells co-stained with the MitoTracker Green FM and MitoTracker™ Red CMXRos for flow cytometry analysis. (**H**) Green fluorescence peak in flow cytometry of N2A cells from the shCTR, Aβo+shCTR, and Aβo+shAGK groups. (**I**) Quantification of the average red fluorescence to green fluorescence ratio, representing relative membrane potential, in N2A cells from shCTR, Aβo+shCTR, and Aβo+shAGK groups, n=3,3,4. (**J**) Quantification of mtDNA relative content, normalized to nDNA, in N2A cells of shCTR, Aβo+shCTR, and Aβo+shAGK groups,n=5,6,6. The graph reports mean±SD; * P < 0.05, ** P < 0.01, *** P < 0.001, **** P < 0.0001(Student’s t-test).


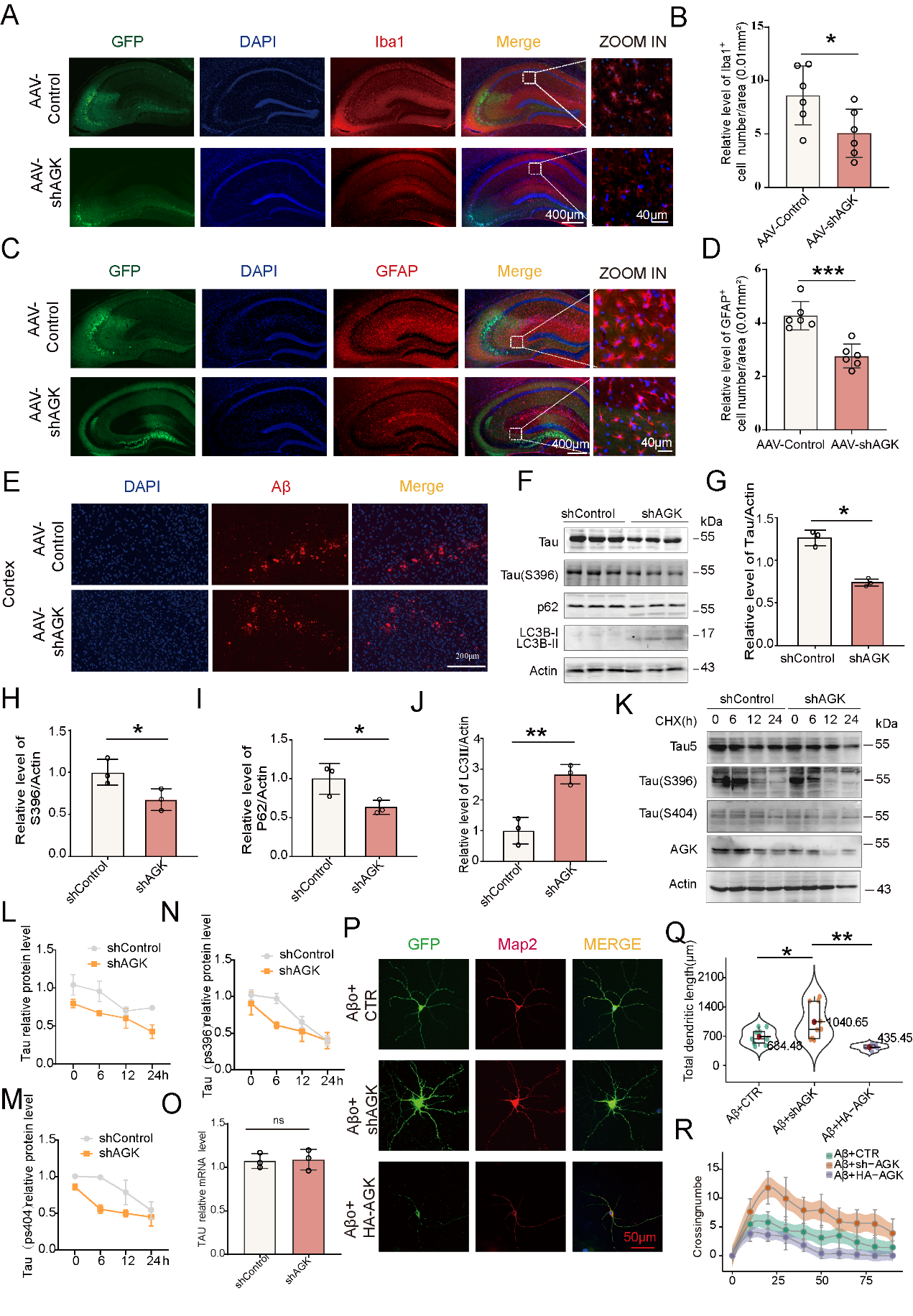


**Figure S5.** Downregulation of AGK decrease the active of microglia and astrocyte.

**(A)(B)** Immunofluorescence staining results for Iba1 in APP/PS1 mice that underwent injection of AAV9-Control and AAV9-shAGK. Scale bar = 400 μm, n=6. **(C)(D)** Immunofluorescence staining results for GFAP for APP/PS1 mice that underwent, Scale bar = 400 μm, n=6. **(E)** Immunofluorescence staining results for Aβ for APP/PS1 mice in cortex that underwent. Scale bar = 200 μm (**F**) Protein immunoblot analysis was performed in AGK-silenced SH-SY5Y cells. (**G**, **H, I and J**) Statistical graphs representing Tau, pS396, LC3, and SQSTM1/p62 protein levels normalized to Actin as in (**F**), n = 3, 3. (**K**) Western blot analysis was used to evaluate protein expression in AGK-silenced SH-SY5Y cells following 400 µg/mL CHX treatment at the indicated time points. (**L**, **M and N**) Statistical graphs representing Tau, pS396, pS404 protein levels normalized to Actin as in (**K**), n=3, 3. (**O**) qPCR analysis of TAU mRNA level was performed in AGK-silenced SH-SY5Y cells, n=3, 3. **(P)** Mouse primary hippocampal neurons were treated with AAV-GFP vector (control), AAV-GFP-shAGK, or AAV-GFP-AGK for 4 days. Representative images are shown. Scale bar = 50 μm. **(Q)** Quantitative analyses of dendritic length of the neurons, n=9,9,5. **(R)** Sholl analysis of the neurons, n=9,9,5. The graph reports mean ± SD * P < 0.05, ** P < 0.01. The graphs are from 3 mouses per group (**B and D**).


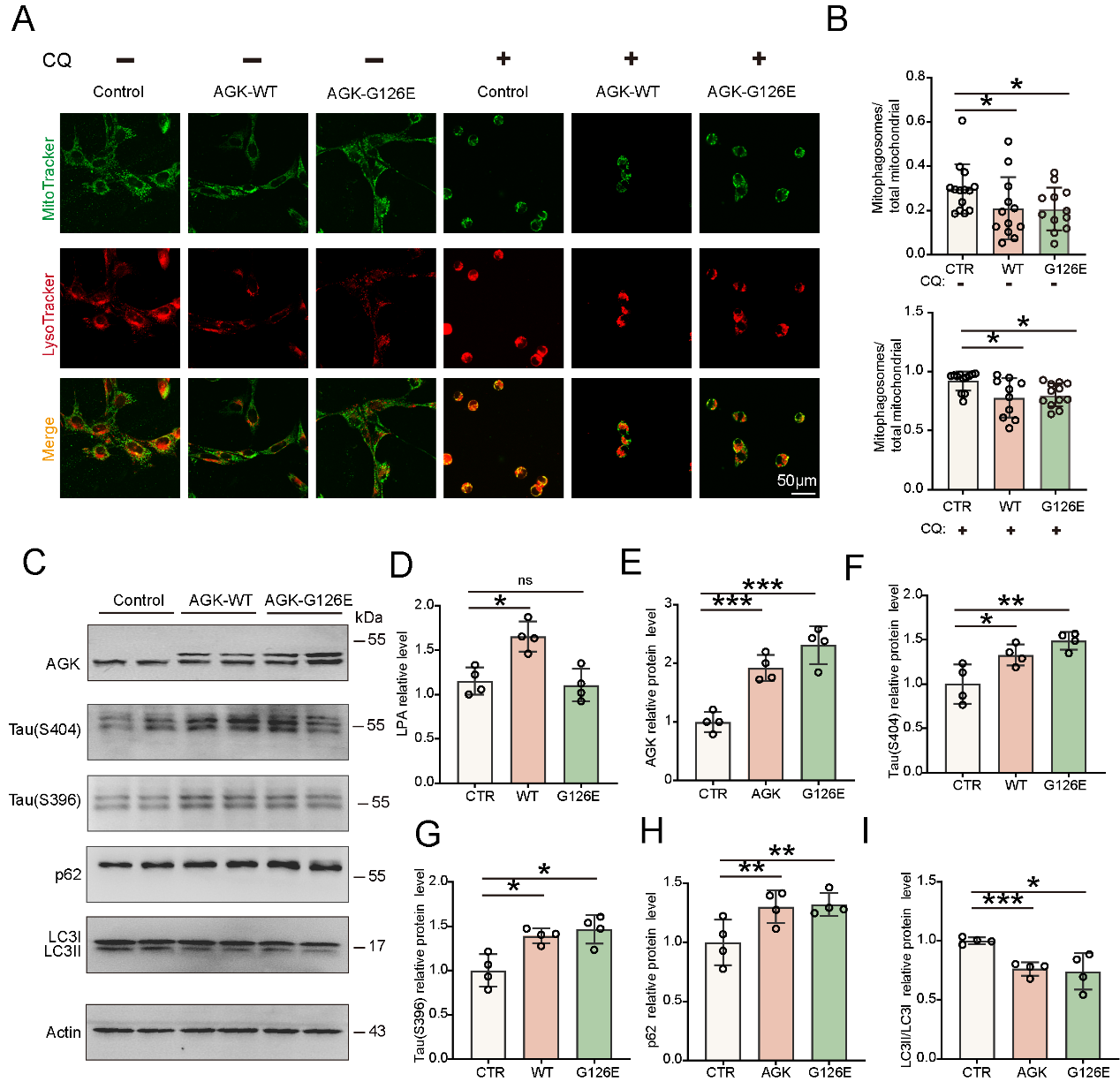


Fig S6 AGK regulates mitophagy and Tau pathology independent of its lipid kinase activity.

(A) Representative confocal images showing colocalization of mitochondria (MitoTracker) and lysosomes (LysoTracker) in N2A cells transfected with control vector, AGK-WT, or kinase-dead AGK G126E mutant, with or without chloroquine (CQ) treatment. Scale bar, 50 μm. (B) Quantification of colocalization of mitochondria and lysosomes in cells expressing AGK-WT or AGK-G126E without chloroquine (CQ) treatment (up), n=14,12,12. Quantification of MitoTracker/LysoTracker colocalization in cells expressing AGK-WT or AGK-G126E with chloroquine (CQ) treatment (down)， n=12,10,12. (C) Representative western blots showing HA-tagged AGK expression, Tau phosphorylation at Ser404 and Ser396, and total p62 and LC3 in N2A cells transfected with control, AGK-WT, or AGK-G126E treated with CQ. (D) Quantification of LPA levels by ELISA, n=4, 4. (E) Quantification of AGK levels, n=4, 4. (F) Quantification of p-Tau Ser404 levels, n=4, 4. (G) Quantification of p-Tau Ser396 levels, n=4, 4. (H) Representative western blots showing autophagy markers p62 and LC3-II/ LC3-I, n=4, 4. (I) Quantification of LC3-II/ LC3-I ratio from (C), n=4, 4. The graph reports mean ± SD; * P < 0.05, ** P < 0.01, *** P < 0.001 (two-tailed Student’s *t*-test).


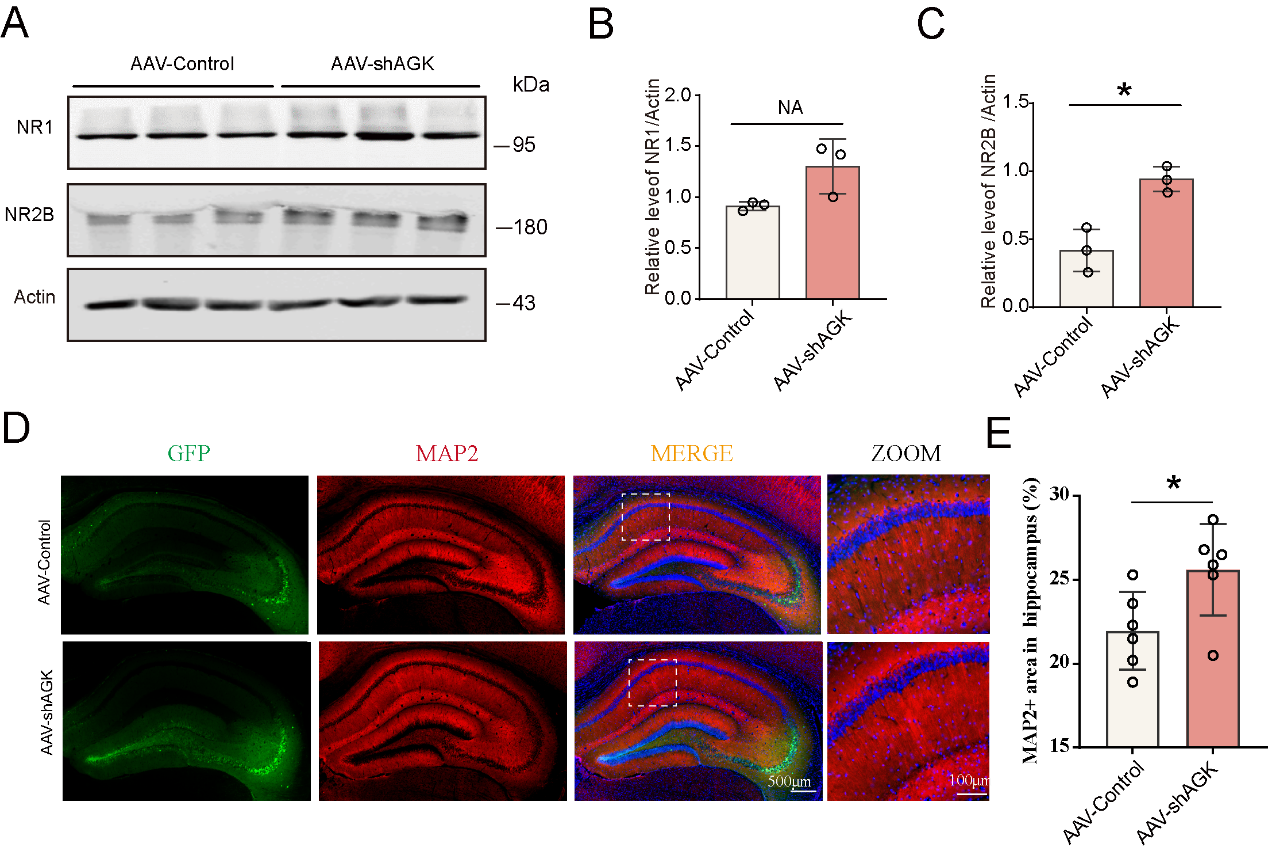


**Figure S7.** Downregulation of AGK rescued synaptic impairments in APP/PS1 mice.

(**A**) Hippocampal tissues from 6-month-old APP/PS1 mice injected with AAV9-Control and AAV9-shAGK viruses were analyzed by immunoblotting. (**B and C**). Graphs representing the protein levels of NR1 and NR2B, normalized to Actin. (n = 3, 3). **(D) (E)** Immunofluorescence staining results for MAP2 in APP/PS1 mice that underwent injection of AAV9-Control and AAV9-shAGK. Scale bar = 500 μm and 100 μm (zoom in), n=6, 6. *: P < 0.05 (two-tailed Student’s *t*-test).

##### Supplementary Tables

**Table S1.** Antibodies used in this study

| Antibodies | Type | Dilution | Souses |
| --- | --- | --- | --- |
| Tau | mAb | 1:1000 | Zen-bioscience (R25862) |
| PINK1 | pAb | 1:1000 | Abcam (ab23707) |
| ATAD3A | pAb | 1:1000 | Abnova (H00055210-D01) |
| Tau(Ser202/Thr205) | mAb | 1:1000 | Zen-bioscience (R23320) |
| Thr181 | mAb | 1:1000 | Zen-bioscience (R23342) |
| Tau(Ser396) | mAb | 1:1000 | Zen-bioscience (381213) |
| Tau (Ser404) | mAb | 1:1000 | Zen-bioscience (R23326) |
| AGK | pAb | 1:1000 | Abcam (ab137616) |
| SQSTM1/p62 | pAb | 1:1000 | Abclonal (A19700) |
| TOM40 | pAb | 1:500 | Santa Cruz (sc-365467) |
| TOM40 | pAb | 1:500 | Abcam (ab185543) |
| TIM23 | pAb | 1:500 | Santa Cruz (sc-514463) |
| LC3 | pAb | 1:1000 | Abclonal (A15591) |
| LAMP2A | pAb | 1:500 | Abcam (ab18528) |
| β-Actin | mAb | 1:5000 | Abclonal (AC006) |
| AT8 | mAb | 1:200 | ThermoFisher (MN1020) |
| Iba1 | mAb | 1:200 | [Cell Signaling Technology](https://www.cellsignal.cn/science-resources/hallmarks-of-ndg/microglial-markers) (17198T) |
| GFAP | mAb | 1:500 | [Cell Signaling Technology](https://www.cellsignal.cn/science-resources/hallmarks-of-ndg/microglial-markers) (3670T) |
| Abeta | mAb | 1:200 | [Cell Signaling Technology](https://www.cellsignal.cn/science-resources/hallmarks-of-ndg/microglial-markers) (8243T) |
| IRDye® 800CW Goat anti-Mouse IgG Secondary Antibody | anti-Mouse IgG | 1:800 | LICOR biosciences(C50133-06) |
| IRDye® 800CW Goat anti-Rabbit IgG Secondary Antibody | anti-Rabbit IgG | 1:800 | LICOR biosciences(C50331-05) |

**Table S2.** Demographics of nondemented control and AD brain tissues used in this study

| Sample | Age | Sex | Braak stage | Brain region | Genotype |
| --- | --- | --- | --- | --- | --- |
| CTR-1 | 83 | Male | N/A | Hippocampus | N/A |
| CTR-2 | 70 | Female | N/A | Hippocampus | N/A |
| CTR-3 | 72 | Male | N/A | Hippocampus | N/A |
| CTR-4 | 86 | Male | N/A | Hippocampus | N/A |
| CTR-5 | 98 | Female | N/A | Hippocampus | N/A |
| CTR-6 | 83 | Female | N/A | Hippocampus | N/A |
| AD-1 | 78 | Male | 4 | Hippocampus | N/A |
| AD-2 | 71 | Female | 4 | Hippocampus | N/A |
| AD-3 | 73 | Male | 4 | Hippocampus | N/A |
| AD-4 | 79 | Male | 6 | Hippocampus | N/A |
| AD-5 | 99 | Female | 5 | Hippocampus | N/A |

**Table S3.** Primers used in this study.

| type | species | DNA | Forward | Reverse |
| --- | --- | --- | --- | --- |
| mRNA | Human | MAPT | *5′-GCTGCTCAGCTCCACATGCATAGTA-3′* | *5′-GGCACCACACAGGCCACACG-3′* |
| mtDNA | Human | ND1： | 5′-*CACCCAAGAACAGGGTTTGT*-3′ | 5′-*TGGCCATGGGTATGTTGTTAA*-3′ |
| mtDNA | Human | D-LOOP | 5′-*CTATCACCCTATTAACCACTCA*-3′ | 5′-*TTCGCCTGTAATATTGAACGTA*-30′ |
| mtDNA | Human | COX3 | 5′-*AATCCAAGCCTACGTTTTCACA*-3′ | 5′-*TGGCCATGGGTATGTTGTTAA*-3′ |
| nDNA | Human | TERT | 5′-*TCACGGAGACCACGTTTCAAA*-3′ | 5′-*TTCAAGTGCTGTCTGATTCCAAT*-3′ |
| mtDNA | Mouse | D-LOOP | 5′-*AATCTACCATCCTCCGTGAAACC*-3′ | 5′-*TCAGTTTAGCTACCCCCAAGTTTAA*-3′ |
| nDNA | Mouse | TERT | 5′-*CTAGCTCATGTGTCAAGACCCTCTT*-3′ | 5′-*GCCAGCACGTTTCTCTCGTT*-3′ |
